# GATA6-AS1 suppresses epithelial–mesenchymal transition of pancreatic cancer under hypoxia through regulating SNAI1 mRNA stability

**DOI:** 10.1186/s12967-023-04757-5

**Published:** 2023-12-06

**Authors:** Yunhui Zhou, Xinyi Zhou, Qiwen Ben, Ningning Liu, Jiahui Wang, Yongpeng Zhai, Yichen Bao, Lin Zhou

**Affiliations:** 1 Department of Gastroenterology, The First Affiliated Hospital of Zhengzhou University, No. 1, Jianshe East Road, Zhengzhou, 450052 China; 2 Department of Gastroenterology, Ruijin Hospital, Shanghai Jiaotong University School of Medicine, Shanghai, 200025 China

**Keywords:** Hypoxia, GATA6-AS1, *N*6-Methyladenosine, Epithelial–mesenchymal transition, Pancreatic ductal adenocarcinoma

## Abstract

Pancreatic ductal adenocarcinoma (PDAC) is characterized by a hypoxic microenvironment, a high rate of heterogeneity as well as a high likelihood of recurrence. Mounting evidence has affirmed that long non-coding RNAs (lncRNAs) participate in the carcinogenesis of PDAC cells. In this study, we revealed significantly decreased expression of GATA6-AS1 in PDAC based on the GEO dataset and our cohorts, and showed that low GATA6-AS1 expression was linked to unfavorable clinicopathologic characteristics as well as a poor prognosis. Gain- and loss-of-function studies demonstrated that GATA6-AS1 suppressed the proliferation, invasion, migration, and epithelial–mesenchymal transition (EMT) process of PDAC cells under hypoxia. In vivo data confirm the suppressive roles of GATA6-AS1/SNAI1 in tumor growth and lung metastasis of PDAC. Mechanistically, hypoxia-driven E26 transformation-specific sequence-1 (ETS1), as an upstream modulatory mechanism, was essential for the downregulation of GATA6-AS1 in PDAC cells. GATA6-AS1 inhibited the expression of fat mass and obesity-associated protein (FTO), an *N*6-methyladenosine (m6A) eraser, and repressed SNAI1 mRNA stability in an m6A-dependent manner. Our data suggested that GATA6-AS1 can inhibit PDAC cell proliferation, invasion, migration, EMT process and metastasis under hypoxia, and disrupting the GATA6-AS1/FTO/SNAI1 axis might be a viable therapeutic approach for refractory hypoxic pancreatic cancers.

**Supplementary Information:**

The online version contains supplementary material available at 10.1186/s12967-023-04757-5.

## Introduction

Hypoxia is a prevalent feature among solid tumors and facilitates the progression of several malignancies [1]. Pancreatic ductal adenocarcinoma (PDAC) is among the most prevalent fatal solid tumors in both sexes in the US, with a 5-year relative survival of 8% or lower [2, 3]. Increasing evidence affirms that hypoxia has a central function in stimulating the epithelial–mesenchymal transition (EMT), which is fundamental for PDAC invasion, metastasis, and therapy resistance [4, 5]. A core set of EMT-activating transcription factors (EMT‐TFs) are involved in the execution of the EMT program and these sets include SNAIL (also SNAI1) and SLUG (also SNAI2), the fundamental helix–loop–helix factors (TWIST1) and TWIST2 and the zinc finger E‐Box binding homeobox factors ZEB1 and ZEB2 [6]. To date, the mechanisms underlying the involvement of hypoxia in EMT as well as progression in PDAC are yet elucidated.

Long noncoding RNAs (lncRNAs) are RNA transcripts that contain greater than 200 nucleotides (nt) with a limited coding capacity [7]. Mounting research has denoted that lncRNAs are abnormally expressed in human malignancies and have a principal function in tumor EMT and invasion. GATA6 antisense 1 (GATA6-AS1) is a lncRNA divergently transcribed from the GATA6 locus. Dysregulated GATA6-AS1 has been reported in non-small cell lung carcinoma (NSCLC) [8], and its abnormal expression is linked to poor prognosis in these individuals. Nonetheless, an in-depth description of the function, as well as the mechanism of GATA6-AS1 in PDAC cells, remains lacking.

Methylation modifications that lead to *N*6-methyladenosine (m6A) are the prevalent abundant internal chemical modifications of RNAs in eukaryotes. M6A modification can affect mRNA localization, export, stability, translation, and splicing [9, 10]. In mammal cells, m6A modification can be reversed and undergoes catalyzation by methyltransferases (methyltransferase-like 3 (METTL3), METTL14, and Wilms tumor 1-associated protein [WTAP]) and demethylases (fat mass and obesity-associated protein [FTO] and alkB homolog 5 [ALKBH5]), also known as “writers” and “erasers”, respectively [11, 12]. Additionally, several different m6A reader proteins, including YT521-B homology (YTH) domain-containing proteins (YTHDF1-3, YTHDC1-2) and IGFBPs can specifically recognize m6A modification to influence RNA function [13].

In this investigation, we manifest that GATA6-AS1 inhibited hypoxia-induced PDAC invasion and EMT in vitro as well as in vivo. We discovered that the expression of GATA6-AS1 is directly inhibited by ETS1 at the transcriptional levels under hypoxia. Importantly, overexpression of GATA6-AS1 might downregulate the expression of FTO under hypoxia, thus substantially decreasing SNAI1 expression in an m6A-dependent pattern.

## Materials and methods

### Cell culture

Human PDAC cell lines, Capan-2, Panc-1, Patu8988T, AsPC-1, SW1990, and BxPC 3, were collected from the Cell Bank of the Chinese Academy of Sciences CAS (Shanghai, China). All cell lines were kept in Dulbecco’s modified Eagle medium (DMEM; Gibco) that contained 10% fetal bovine serum (FBS; Gibco) as well as 1% penicillin-streptomycin. Human normal pancreatic epithelium cell line HPNE, supplied by American Type Culture Collection, was incubated in the DMEM/F12 medium (Gibco) that contained 0.5 µg/ml hydrocortisone, 5% horse serum, 20 ng/ml epidermal growth factor, 2 mM l-glutamine, 10 µg/ml insulin, and 0.1 µg/ml cholera enterotoxin. The cell lines were subjected to authentication through a short tandem repeat (STR) profiling analysis in the Cell Bank of Type Culture Collection of CAS (Shanghai, China). All cells were mycoplasma-free confirmed with the Universal Mycoplasma Detection Kit (ATCC, Manassas, VA, USA). To achieve the hypoxic condition, the cultured cells were flushed with 1% O_2_, 5% CO_2_, and 94% N_2_, or 5% O_2_, 5% CO_2,_ and 90% N_2_ mixture gas in a hypoxia chamber.

### lncRNA expression microarray analysis

Microarray analysis was conducted as a previous report [14]. In brief, total RNA from Panc-1 cells treated with 1% or 20% O_2_ was extracted using TRIzol reagent (Invitrogen, Thermo Fisher Scientific). Next, cDNA was synthesized using 0.5 µg total RNA via a GeneChip Transcription Express Kit (Thermo Fisher Scientific Inc., Waltham, MA, USA). The microarray was then scanned using an Agilent G2505B Microarray Scanner (Agilent Technologies).

### Plasmids, short hairpin RNAs, and cell transfection

To construct the expression vector, cDNAs encoding full-length of GATA6-AS1, ETS1, FTO, and SNAI1 were subcloned into the pcDNA3.1 (+) vector (Invitrogen, Carlsbad, CA). Short hairpin RNA (shRNA) against GATA6-AS1, ETS1, FTO, METTL3, METTL14, WTAP, ALKBH5, YTHDF2, and SNAI1, as well as shRNA negative control (shNC), were designed and purchased from Genepharma (Shanghai, China). For RNA investigation, at least two independent shRNA sequences were evaluated for every gene. Sequences for shRNAs involved are outlined in Additional file 1: Table S1. All products were validated by DNA sequencing. When reaching 70–80% confluence, the cells were transiently transfected utilizing Lipofectamine 3000 reagent (Invitrogen, Carlsbad, California, USA) as per the manufacturer’s specifications.

### Lentivirus synthesis and transduction

A lentiviral vector expressing either a full-length GATA6-AS1 or SNAI1 or shRNA targeting GATA6-AS1 was synthesized and subcloned into the lentiviral vector. Centrifugation was instrumental in concentrating recombinant lentiviruses, and we then dissolved them in DMEM and kept them at − 80 °C for subsequent use. These lentivirus vectors were transfected into PDAC cells (3 × 10^5^/well) with polybrene (5 µg/ml, Sigma-Aldrich) according to the manual. The infected cells were thereafter chosen with 2 mg/l puromycin for 14 days.

### Human PDAC tissue specimens

This study employed 2 distinct cohorts of human PDAC tissues. The formalin-fixed and paraffin-embedded (FFPE) PDAC tissue containing 116 pairs of PDAC tissues (T) and adjacent nontumor tissues (N) were utilized to create a tissue microarray (TMA) (cohort A) for in situ hybridization (ISH) and immunohistochemical (IHC) analysis. The fresh PDAC tissue samples containing 72 pairs of T and N tissues were used for the RT-qPCR assay (cohort B). Cohort A was gathered from Ruijin Hospital, Shanghai Jiaotong University School of Medicine (Shanghai, China) from January 2014 to May 2016 and cohort B from the First Zhengzhou University (Zhengzhou, China) from April 2016 to December 2019. Demographic characteristics and clinical information of individuals were collected from the medical records. Regarding these patients, none of them was subjected to chemotherapy or radiotherapy before surgery. The study protocol was performed according to the ethics committee of the chamber of physicians of Shanghai Jiaotong University School of Medicine, and Zhengzhou University, China. Informed consent was available from all patients.

#### Scoring

ISH assay of GATA6-AS1 was performed using a double digoxigenin (DIG)-tagged mercury locked nucleic acid (LNA) probe (miRCURY LNA™, Exiqon, Denmark). The GATA6-AS1 probe sequence used is shown below: 5′ Dig-ACTCACAGTTACGTGCAGAGGA-Dig 3′. For the IHC assay of E-cad, Vim, and SNAI1, the DAKO Envision system (DAKO, Carpinteria, California) was used as described earlier [15]. After quenching endogenous peroxidase action and preventing nonspecific attachment, the slides were treated overnight at 4 °C with the specified antibody or probes. The staining intensity was graded utilizing a scale of 0–3 (0, negative; 1, weak; 2, moderate; 3, strong) and the staining range was assessed according to the percentage of staining in five random fields (1, 0–10%; 2, 10–50%; 3, 50–75%; 4, > 75%). The eventual score was derived by multiplying both scores and was utilized to categorize the samples into 3 grades: weak staining (score 0–3); medium staining (score 4–6); and strong staining (score 7–12). When conducting survival analyses, we integrated weak and medium staining as a low expression (score 0–6), whereas strong staining was regarded as a high expression (score 7–12). Two pathologists who were blinded to the clinical data graded all sections independently.

### RNA isolation and real-time quantitative PCR analysis

Total RNA was extracted from fresh pancreas tissues and cell lines utilizing TRIzol reagent (Invitrogen, Carlsbad, CA, USA) following the manufacturer’s specifications. We used the SYBR® Premix Ex Taq kit (Takara Bio Inc., Shiga, Japan) to determine the relative RNA level, which was derived utilizing the comparative Ct method. The specific primer sequences were shown Additional file 1: Table S2 and were synthesized and purified by the Shanghai GenePharma Co. (Shanghai, China). β-Actin was served for normalization. The BLAST algorithm (National Center for Biotechnology Information) was instrumental in the verification of the sequence specificity.

#### Methylation-specific polymerase chain reaction

The genomic DNA of PDAC cells was isolated using a commercial kit (TIANGEN, Beijing, China), and a DNA Methylation-Gold™ kit (D5005, Zymo Research, Irvine, CA) was exploited to assess the methylation level of FTO promoter. Methylated (M) and unmethylated (U) primers were displayed in Additional file 1: Tables S1, S2. The PCR products were then electrophoresed with 3% agarose gel, and the target bands were observed by gel imager. The experiment was repeated three times.

### Cytoplasmic and nuclear fractionation

Nuclear and cytoplasmic fractionation from PDAC cells was executed using PARIS Kit (Life Technologies, Carlsbad, CA) and assessed by RT-qPCR detection. U6, as well as β-Actin, were taken as the nuclear and cytoplasmic controls, correspondingly.

### RNA stability analysis

On a 12-wells plate, PDAC cells with transfection of the indicated vectors were seeded and kept in an incubator overnight at 37 °C. Stability of RNA was conducted with 5.0 µg/ml of Actinomycin D (Act-D, Sigma, U.S.A) at the stipulated times. Total RNA was obtained with the help of TRIZOL reagent (Invitrogen) and underwent RT-qPCR analysis. β-Actin served as the internal control. The half-life of mRNA was estimated based on previous study [16]. The rate of disappearance of mRNA concentration at a given time (dC/dt) is proportional to both the rate constant for decay (Kdecay) and the cytoplasmic concentration of the mRNA (C). This relation is described by the following equation: dC/dt = − KdecayC. The mRNA decay rate (Kdecay) was estimated as ln(C/C0) = − Kdecay t. Thus, the half-life (t1/2) was estimated by the following equation: t1/2 = ln2/Kdecay.

### Protein extraction and Western blot assays

Total protein was prepared from cells and tissues utilizing detergent-containing lysis buffer, and the concentration of protein was ascertained utilizing a bicinchoninic acid assay kit (Bio-Rad Laboratories). Total protein (25 µg) received SDS-polyacrylamide gel separation and was transferred to PVDF membrane (Millipore). Primary antibodies against: ETS1 (ab238645, Abcam), hypoxia-inducible factor 1 alpha (HIF1A, ab51608, Abcam) and 2 alpha (HIF2A, ab243861, Abcam), E-cad (ab40772, Abcam), Vim (ab8069, Abcam), ZEB1 (ab245283, Abcam), TWIST1 (CST, 69366S, Abcam), FTO (ab126605, Abcam), SNAI1 (sc-271977, Santa Cruz), DNMT1 (ab19905, Abcam), DNMT3A (ab188470, Abcam), DNMT3B (ab2851, Abcam), and METTL3 (ab195352, Abcam) were used. The protein loading control was β-actin. Quantifications of Western blots were analyzed using Image J V1.53c (National Institutes of Health).

#### In vitro proliferation, invasion, and migration assays

Onto a 96-well plate cells were seeded (2000 cells per well). Afterward, upon attaining a predetermined time of culture, cell viability was ascertained utilizing MTT assays. The optical density (OD) was taken at 450 nm in every well using a microplate reader (BioRad, Hercules, CA, USA). For the EdU assay, the Yefluor 594 Edu Imaging Kits (Yeason, China) were adopted following the manufacturer’s protocols. Transfected cells were subjected to culturing with Edu for 2 h. Then, they were fixated with 4% paraformaldehyde, stained with Yefluor 594 Azide Solution, and ultimately mounted with DAPI (Sigma-Aldrich, St. Louis, Missouri, USA). EdU positive cells were reported as the quantity of EdU (red) positively stained cells/the quantity of DAPI (blue) positively stained cells in three randomly picked fields. On the other hand, a pipette tip was utilized in the wound-healing assay to create horizontal streaks in the cells that were grown in the 12 well plates until they attained confluence. After 24 h, photos were retaken, and the distance of migration was measured at 0 and 24 h. About invasion assays, the 8-µm pore inserts were coated with 30 µg of Matrigel (BD Biosciences). PDAC cells (2 × 10^5^) were placed in the top chambers in a medium that was free of serum. As a chemoattractant, DMEM medium having 20% FBS was added to the bottom chambers. Upon incubation for 24 h at a temperature of 37 °C, the invaded cells were fixed, stained utilizing crystal violet, and observed using a microscope at 200× magnification for enumeration in three randomly picked fields. The migration assay was performed in a similar method devoid of coating the filters utilizing Matrigel.

### Tumor xenografts

Female athymic BALB/c mice, aged 4–5 weeks, were procured from Slack, Shanghai, China, and were fed under the standard pathogen-free settings. Logarithmic phase PDAC cells (4 × 10^6^/100 µl) were infected using lentiviruses possessing constructs. Then, the nude mice (n = 5 per group) were inoculated into the dorsal flank subcutaneously. The animals under investigation were thoroughly monitored, and the tumor size was measured at an interval of 5 days. The volume of the tumor was ascertained using the equation below: volume (mm^3^) = length × width^2^ × 0.5. Mice were killed utilizing CO_2_ as per the animal welfare specifications. All animal studies were approved by the Institutional Animal Care and Use Committee of Zhengzhou University (Henan, China). For the metastatic lung model, a total of 1 × 10^6^ cells of SW1990 cells stably-transfected with sh-GATA6-AS1#1, #2 or control vector were injected into tail veins of BALB/c nude mice. After all these mice were sacrificed, the tumors and lung were excised and processed for analysis.

### LncRNA fluorescence (FISH) and immunofluorescence staining

RNA FISH was conducted on PDAC cells and tissues utilizing RNA Fluorescence In Situ Hybridization Kit (Exonbio Lab, Guangzhou, China). RNA FISH, as well as immunofluorescence staining, were used to ascertain the colocalization of *GATA6-AS1* in cells. Fluorescein-labeled RNA probes for GATA6-AS1 were utilized to hybridize the PDAC cells overnight at 37 °C. Slides were washed and thereafter counterstained with 4′-6′diamidino-2-phenylindole (DAPI, Beyotime). For immunofluorescence (IF) staining, the tissue slices were fixed in 4% paraformaldehyde for 30 min, permeabilized, and blocked utilizing 5% bovine serum albumin (BSA) (Sigma, St Louis, MO) for 1 h. After the tissue sections underwent incubation with primary antibody against E-cad and Vim, and secondary fluorescent antibody (Invitrogen, 594 nm), respectively, the tissue sections were subjected to DAPI for 10 min to stain them. Finally, fluorescence images were obtained by confocal microscope (Olympus FV1000, Tokyo, Japan).

#### Chromatin immunoprecipitation

As described previously [17], chromatin immunoprecipitation (ChIP) assays were carried out utilizing the EZ ChIP Kit (Millipore, MA, USA). The proteins were cross-linked to the DNA by adding 1% formaldehyde at RT for 15 min, and the DNA was split to a mean fragment size of 200–800 bp by sonication. After DNA-protein samples were precleared with protein A/G beads, they were exposed to immunoprecipitation overnight with an anti-ETS1 antibody or IgG. Next, the related genomic DNA was heated to reverse histone-DNA crosslinks and was assessed by qPCR with GATA6-AS1 promoter-specific primers.

### RNA immunoprecipitation

RNA immunoprecipitation (RIP) assay was conducted with the utilization of the Magna RIP Immunoprecipitation kit (Millipore, MA) based on the manufacturer’s specifications. After washing with PBS twice, 1 × 10^7^ PDAC cell lines were lysed in a *c*omplete RIP lysis buffer. After centrifuging and removing the cell debris, the supernatant was retained as input, and the whole cell extract was co-immunoprecipitated with an antibody against FTO, METTL3, METTL14, WTAP, and ALKBH5 or control IgG at 4 °C overnight. The enrichment of RNA was determined by RT-qPCR.

### Luciferase reporter gene assays

The GATA6-AS1 promoter regions having two ETS1 putative binding areas (wild type, WT) or two mutant areas (mutant type: MUT1 or MUT2) were respectively introduced into the pGL3 vector (Promega, Madison, WI), and then was transfected into 293 T cells. To determine the effects of FTO on SNAI1 mRNA containing potential two m6A sites (supported by SRAMP prediction) or mutant type (adenosines in m6A positions were replaced by cytosines) were inserted into luciferase reporter vectors. Relative luciferase activity was calculated by “F-luc/R-luc” utilizing the Dual-Luciferase Reporter Assay System (Promega).

### RNA pull-down assay

Biotin-labeled RNAs, including FL (full-length), Antisense and serial truncations of GATA6-AS1, were transcribed in vitro with the Biotin RNA Labeling Mix and T7 RNA polymerase (Roche, Basel, Switzerland). Purified biotin-labeled RNA was heated and annealed to form a secondary structure, mixed with streptavidin agarose beads (Life Technologies, Gaithersburg, MD) at 4 °C for 1 h. Total cell lysates were freshly prepared and added to each binding reaction with Protease/Phosphatase Inhibitor Cocktail and RNase inhibitor. Finally, the RNA-binding proteins were analyzed by Western blot.

### Methylated RNA immunoprecipitation (MeRIP) assay

The Magna MeRIP m6A Kit (Millipore, USA) was employed in determining the enrichment of m6A at particular sites on the SNAI1 transcript. After total RNA was extracted from PDAC cells and tissues, total RNA (1 µg/µl) was sheared to fragmentation about 100 nt in length. Thereafter, the fragmented RNA underwent incubation with an anti-m6A antibody (1 µg) or control IgG-conjugated Dynabeads in an IP buffer for 4 h with rotation. Ultimately, the co-precipitated RNA specimens having m6A modification sites were utilized for qPCR determination. Certain primers were designed based on the m6A modification sites on the SNAI1 transcript with greater confidence (anticipated by SRAMP) [18]. Additional file 1: Table S1 lists the primer sequences.

### Bioinformatics analysis

RNAseq data were retrieved from two independent PDAC cohorts, GSE15471 and The Cancer Genome Atlas (TCGA)-Pancreatic adenocarcinoma (TCGA-PAAD) databases, correspondingly. GSE15471 has 39 pairs of PDAC malignant tissues as well as normal tissues. TCGA-PAAD contains 178 cancerous samples and 4 normal samples. The potential RNA–protein interactions were anticipated by the machine learning classifier RPISeq [19] utilizing Random Forest (RF) or Support Vector Machine (SVM) classifiers. The prediction of m6A modification sites was performed using the online tools SRAMP [18], depending on epitranscriptome sequencing information as well as machine learning pattern.

### Statistical analysis

Data were presented as means ± SD for at least n = 3 independent experiments except otherwise explanation. Regarding comparisons, the student’s t-test, Pearson chi-square test, and the Wilcoxon signed-rank test were conducted when necessary. Spearman correlation analysis analyzed correlations. Kaplan–Meier plots as well as log-rank tests ascertained overall survival analysis. Univariable and multivariable Cox proportional hazards regression models were instrumental in analyzing independent prognostic factors. *P* < 0.05 was statistically significant. Statistical analyses were carried out by SPSS19.0 (SPSS, Chicago, IL) and GraphPad Prism version 8.0 (GraphPad Inc., La Jolla, CA, USA).

## Results

### Hypoxia inhibits GATA6-AS1 expression in PDAC and downregulated GATA6-AS1 predicts a dismal prognosis

**Fig. 1:**
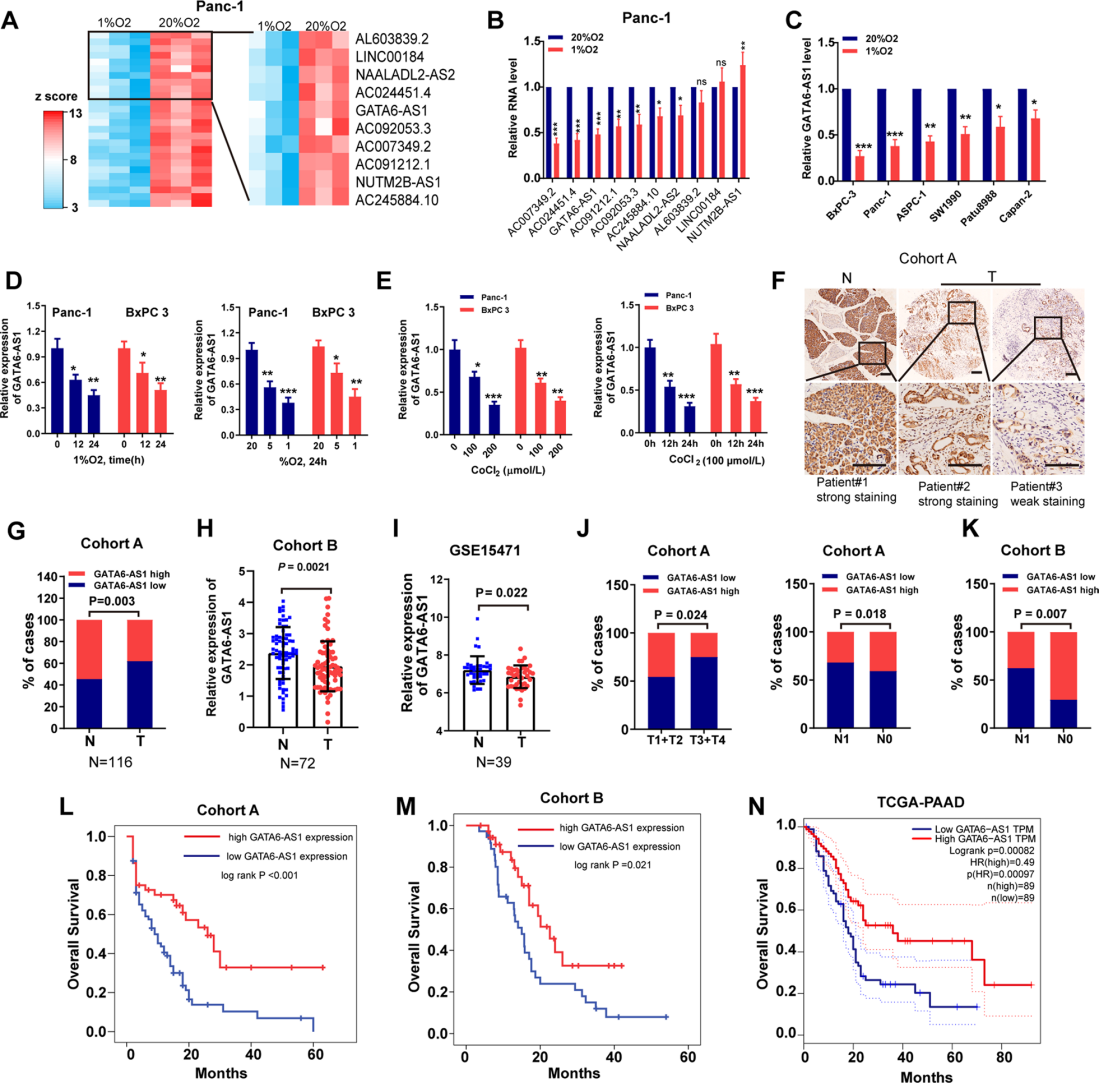
GATA6-AS1 is responsive to hypoxia and its low expression predicts dismal prognosis in PDAC. **A** Heatmap plots of significantly downregulated lncRNAs (*P* < 0.05, fold change > 2 or < 0.5) detected with lncRNA array in Panc-1 cells exposed to 1% O_2_ compared with those exposed to 20% O_2_. The red (higher expression) or blue (lower expression) color represents the normalized expression value of indicated lncRNAs. **B** RT-qPCR assay of expression levels of top 10 downregulated lncRNAs in Panc-1 cells exposed to 1% O_2_ compared with those exposed to 20% O_2_. **C** RT-qPCR assay of expression levels of GATA6-AS1 in PDAC cells exposed to 1% O_2_ compared with those exposed to 20% O_2_. **D** RT-qPCR assay of expression levels of GATA6-AS1 in Panc-1 and BxPC 3 cells exposed to hypoxia conditions at varying time intervals (0, 12, and 24 h) and concentrations of oxygen (1%, 5% or 20%). **E** RT-qPCR assay of expression levels of GATA6-AS1 in Panc-1 and BxPC 3 cells treated with CoCl_2_ for varying dose (0, 100, and 200 µmol/l) or time intervals (0, 12, and 24 h). **F** In situ hybridization assay of GATA6-AS1 expression in PDAC samples and matched adjacent nontumor tissues (Cohort A, n = 116). Scale bar, 200 μm. **G**–**I** GATA6-AS1 was expressed at a lower level in PDAC tumor tissues compared to that in adjacent nontumor tissues from cohort A, B (n = 72), and GSE15471 (n = 39), respectively. **J**, **K** Low GATA6-AS1 expression was more frequently observed in patients with advanced clinical parameters in cohort A (**J**) and cohort B (**K**) of PDAC patients. **L**–**N** Kaplan–Meier curves for overall survival rates associated with GATA6-AS1 expression in our cohort A, cohort B or TCGA-PAAD database. CoCl_2_: cobalt chloride; T: tumor tissues; N: adjacent nontumor tissues. Data represent mean ± S.D. from three independent experiments. *P < 0.05; **P < 0.01; ***P < 0.001

To screen lncRNAs that are responsive to hypoxic conditions, we comparatively analyzed lncRNA profiles using the microarray test for Panc-1 cells exposure to 1% or 20% O_2_ for 24 h. Results found 305 upregulated and 263 downregulated (*P* < 0.05, fold change > 2 or < 0.5) lncRNAs, and we focused on the top 10 downregulated lncRNAs responsive to hypoxia (Fig. 1A). We first tested expression levels of these lncRNAs in Panc-1 cells cultured at 1% or 20% O_2_ for 24 h by RT-qPCR. Of which, GATA6-AS1 was listed in the top 5 downregulated lncRNAs under hypoxia conditions when compared with normoxia, indicating that deregulation of GATA6-AS1 might have a pivotal role in PDAC progression under hypoxia (Fig. 1B). Moreover, the downregulated GATA6-AS1 was verified in other hypoxic PDAC cells (Fig. 1C). To additionally validate its hypoxia-dependence in PDAC cells, we incubated Panc-1 and BxPC 3 cells in hypoxia (1% and 5% O_2_) or normoxia (20% O_2_) for 24 h, or in hypoxia (1% O_2_) or normoxia for 0 h, 12 h, and 24 h, respectively. We found that expression levels of GATA6-AS1 were decreased in hypoxic PDAC cells in a dose- and time-dependent manner (Fig. 1D). Furthermore, for chemically stimulated hypoxia, we treated Panc-1 and BxPC 3 cells with cobalt chloride (CoCl_2_) and found that CoCl_2_ inhibited GATA6-AS1 expression in a concentration- and time-dependent manner (Fig. 1E).

We then performed an ISH assay to measure GATA6-AS1 expression in cohort A of PDAC specimens (Fig. 1F). Generally, GATA6-AS1 was downmodulated in PDAC tissues in contrast with adjacent nontumor tissues according to analysis of specimens from cohort A and B (Fig. 1G, H). These findings were in agreement with the data from the GSE15471 dataset, which included 39 patients with PDAC (Fig. 1I). We then performed an analysis of the link between GATA6-AS1 expression and clinical conditions in cohort A and cohort B of PDAC patients. As demonstrated in Fig. 1J, K, low expression levels of GATA6-AS1 were primarily detected in individuals with advanced T and N stages in cohort A, and with the advanced N stage in cohort B. The further Kaplan–Meier survival analysis showed that low GATA6-AS1 expression was linked to the dismal OS of PDAC individuals from cohort A, cohort B, and TCGA-PAAD (Fig. 1L–N). Univariate and multivariate Cox proportional hazards analysis revealed that GATA6-AS1 was an independent prognostic factor in our two cohorts of PDAC patients (Tables 1 and 2). Furthermore, when comparing PDAC cell lines to the HPNE cell line, the expression levels of GATA6-AS1 were remarkably decreased in the PDAC cell lines (Additional file 1: Fig. S1A). Collectively, these data suggest that deregulated GATA6-AS1 may respond to hypoxia and be involved in PDAC progression.

**Table 1:** Univariate and multivariate Cox regression analyses of overall survival in Cohort A of pancreatic cancer

Parameters	Univariate analysis, HR (95% CI)	P	Multivariate analysis, HR (95% CI)	P value
T stage				
T3–T4 vs. T1–T2	1.98 (1.24–3.15)	0.004	1.86 (1.15–3.00)	0.011
N stage				
N1 vs. N0	1.12 (0.66–1.92)	0.674		
Tumor grade				
G3 vs. G1 + G2	0.94 (0.60–1.47)	0.777		
Tumor size, cm				
≥ 4 vs. < 4	2.11 (1.33–3.36)	0.002	1.53 (0.94–2.50)	0.089
Vascular invasion				
Yes vs. no	1.43 (0.45–4.57)	0.542		
Adjuvant therapy				
Yes vs. no	0.46 (0.28–0.75)	0.002	0.61 (0.37–1.01)	0.056
Neural invasion				
Yes vs. no	2.34 (1.39–3.91)	0.001	2.35 (1.37–4.01)	0.002
GATA6-AS1 staining				
High vs. low	0.43 (0.26–0.71)	0.001	0.66 (0.47–0.93)	0.018

Tumor classification and stage were referred to the 8th edition of UICC on cancer staging system

*HR* hazard ratio, *CI* confidence interval

**Table 2:** Univariate and multivariate Cox regression analyses of overall survival in Cohort B of pancreatic cancer

Parameters	Univariate analysis, HR (95% CI)	P	Multivariate analysis, HR (95% CI)	P value
T stage				
T3–T4 vs. T1–T2	1.23 (0.52–2.87)	0.64		
N stage				
N1 vs. N0	1.67 (0.90–3.07)	0.102	1.53 (0.89–2.52)	0.121
Tumor grade				
G3 vs. G1 + G2	2.31 (1.27–4.20)	0.006	2.27 (1.20–4.30)	0.012
Tumor size, cm				
≥ 4 vs. < 4	1.59 (1.02–2.49)	0.042	1.67 (0.94–2.96)	0.079
Vascular invasion				
Yes vs. no	1.84 (1.02–3.31)	0.041	1.98 (0.88–4.45)	0.098
Neural invasion				
Yes vs. no	1.69 (0.93–3.07)	0.102	2.79 (1.01–7.67)	0.047
GATA6-AS1 expression				
High vs. low	0.49 (0.26–0.91)	0.024	0.57 (0.34–0.96)	0.036

Tumor classification and stage were referred to the 8th edition of UICC on cancer staging system

*HR* hazard ratio, *CI* confidence interval

*GATA6-AS1* locus is located on chromosome 18, next to *GATA6* (Additional file 1: Fig. S1B**)**, an important tumor suppressive transcription factor in PDAC [20]. GATA6-AS1 transcript is characterized 1788 nucleotide long sequence (accession no. NR_102763.1). Like GATA6-AS1, hypoxic conditions led to the downregulated expression GATA6 in PDAC cells in a dose- and time-dependent manner (Additional file 1: Fig. S1C–E). Furthermore, online analyses of CCLE (https://portals.broadinstitute.org/ccle) and TCGA dataset validated a significantly positive correlation between GATA6-AS1 and GATA6 expression in PDAC cell lines and tissues (Additional file 1: Fig. S1F). To investigate the subcellular site of GATA6-AS1, cellular fractionation experiments and LncRNA FISH assay were performed, which exhibited that GATA6-AS1 was not only localized to the cytoplasm but also nucleus, whereas the cytoplasmic distribution was the primary (Additional file 1: Fig. S1G, H). Concurrently, the protein-coding capability of GATA6-AS1 was ascertained by LNCipedia (https://lncipedia.org), which verified GATA6-AS1 as a non-coding RNA (Additional file 1: Fig. S1I). Additionally, the secondary structure of GATA6-AS1 is anticipated by RNAfold Webserver (http://rna.tbi.univie.ac.at/; Additional file 1: Fig. S1J).

### GATA6-AS1 inhibits hypoxia-induced PDAC progression and the EMT process

**Fig. 2:**
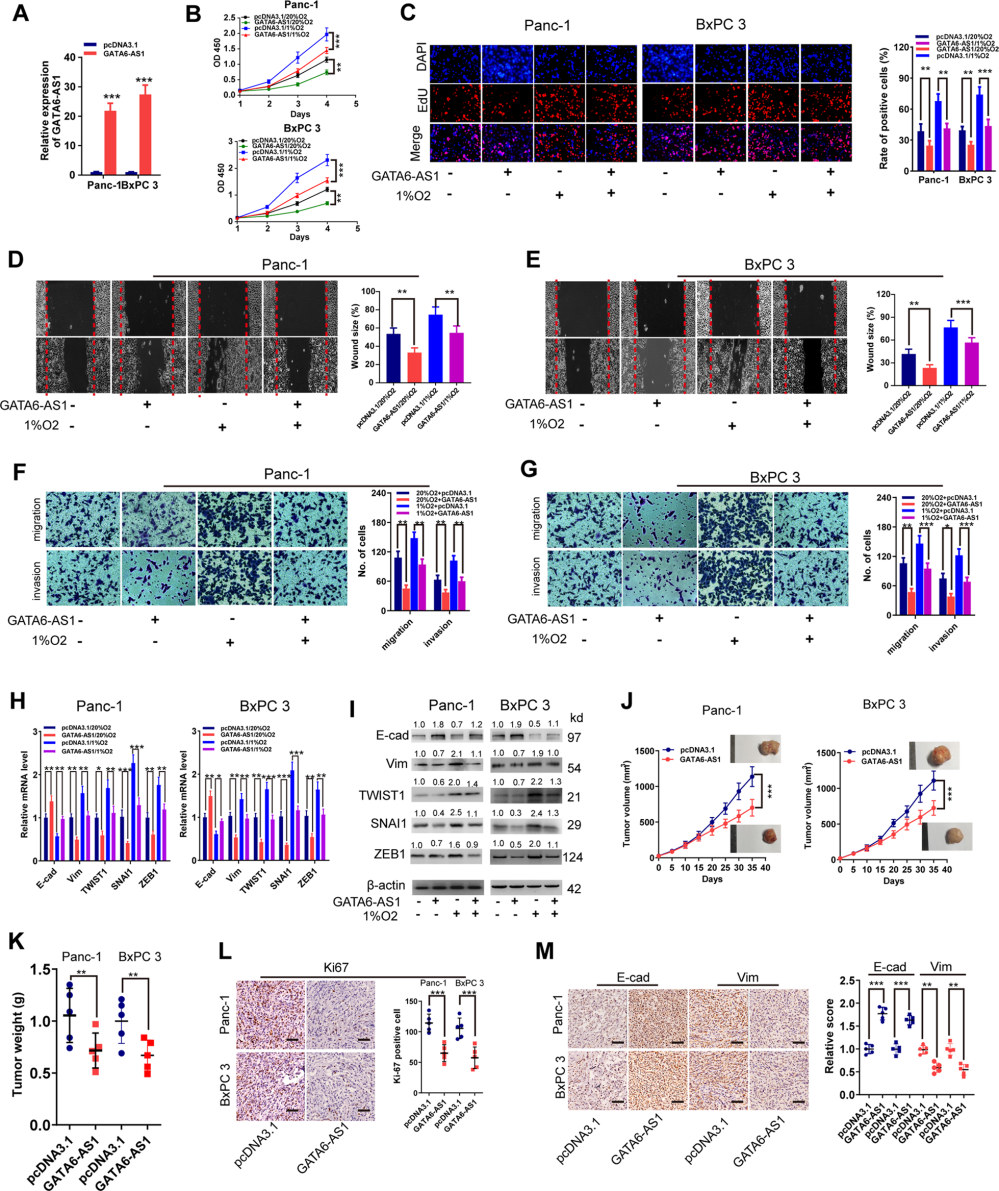
Overexpression of GATA6-AS1 inhibits hypoxia-induced PDAC tumorigenesis and EMT in vitro and in vivo. **A** RT-qPCR and western blot assays analysis of GATA6-AS1 in PDAC cell lines transfected with GATA6-AS1 or pcDNA3.1. **B**–**G** The MTT (**B**), Edu (**C**), wound healing (**D**, **E**) and transwell (**F**, **G**) assay analysis of cell viability, migration and invasion in Panc-1 and BxPC 3 cell lines transfected with GATA6-AS1 or pcDNA3.1 under 1% or 20% O_2_. **H**, **I** RT-qPCR and western blot assays analysis of EMT markers in PDAC cell lines transfected with GATA6-AS1 or pcDNA3.1 under 1% or 20% O_2_. **J**, **K** GATA6-AS1-overexpression or control-transfected Panc-1 and BxPC 3 cell lines (5 × 10^6^ per mouse) were injected into the right dorsal of nude mice. Tumor volume curve and tumor weight of subcutaneous xenografts was analyzed. **L**, **M** The tumor sections were subjected to immunohistochemistry staining using antibodies against ki-67, E-cad and Vim. Scale bar, 100 μm. Data represent mean ± S.D. from three independent experiments. *P < 0.05; **P < 0.01; ***P < 0.001

To investigate the functional role of hypoxia-inhibited GATA6-AS1, we first used full-length GATA6-AS1 to upregulate GATA6-AS1 expression, and RT-qPCR assay revealed an upregulated GATA6-AS1 expression (up to 20-fold) in Panc-1 and BxPC 3 cells (Fig. 2A). We next examined the impacts of GATA6-AS1 on in vitro proliferation, invasion, migration, and EMT process in Panc-1 and BxPC 3 cells cultured in hypoxic (1% O_2_) or normoxic (20% O_2_) parameters. As shown in Fig. 2B–G, overexpression of GATA6-AS1 suppressed PDAC cell proliferation, migration and invasion capacity in hypoxic or normoxic conditions. It is well known that the malignant advancement of multiple types of cancer, including PDAC, relies on EMT activation in cancer cells [5]. We then examined the expression of EMT markers utilizing RT-qPCR as well as western blotting assays. Expectedly, findings demonstrated that transfection of GATA6-AS1 inhibited Vim, TWIST1, SNAI1, and ZEB1 expression levels, but enhanced E-cad levels of Panc-1 and BxPC 3 cells in hypoxic or normoxic conditions (Fig. 2H, I**)**.

These impacts were further validated in vivo with the aid of mouse xenograft models. When compared to each control, subcutaneous xenografts produced from Panc-1 and BxPC 3 cells overexpressing GATA6-AS1 exhibited significantly lower growth rates (Fig. 2J, K). Then, an IHC assay was used to analyze tumor proliferation and showed that tumors from the GATA6-AS1 overexpression groups had decreased expression levels of Ki-67 compared to those from the control group (Fig. 2L). We then examined the expression levels of E-cad, Vim, TWIST1, and SNAI1 utilizing RT-qPCR as well as western blotting assay, and the data exhibited that GATA6-AS1 overexpression led to decreased levels of these markers’ expression (Additional file 1: Fig. S2A). IHC and IF assay confirmed the decreased E-cad, but enhanced Vim expression, in the xenografts derived from overexpressing GATA6-AS1 compared with each control (Fig. 2M and Additional file 1: Fig. S2B). Moreover, we assessed protein levels of E-cad as well as Vim in cohort A of human PDAC tissue by using IHC and analyzing the correlation with GATA6-AS1. The findings verified that expression of GATA6-AS1 was inversely linked to Vim in cancer specimens, but was positively linked to E-cad expression (Additional file 1: Fig. S3A). Additionally, we ascertained that GATA6-AS1 was inversely linked to most EMT markers in PDAC tissues from the TCGA-PAAD dataset and GSE15471 (Additional file 1: Fig. S3B, C). To expand our findings, we transfected three parallel shRNAs targeting GATA6-AS1 (shAS1#1, #2, and #3) into SW1990, and RT-qPCR analysis demonstrated the efficiencies for GATA6-AS1 knockdown in SW1990 cells, especially for shAS1#1 and #2 (Additional file 1: Fig. S4A). Thus, we selected these two shRNAs for the subsequent experiments. The loss of function experiments in vitro and in vivo confirmed that knockdown of GATA6-AS1 strengthened hypoxia-induced cell proliferation, migration, invasion, EMT process (Additional file 1: Fig. S4A–F), tumor growth and lung metastasis (Additional file 1: Fig. S5A–G). Based on these data, we verified that GATA6-AS1 inhibited hypoxia-induced PDAC cell proliferation, migration, invasion, EMT process and metastasis.

### Hypoxia represses GATA6-AS1 expression in PDAC through ETS1 expression at the transcriptional levels

**Fig. 3:**
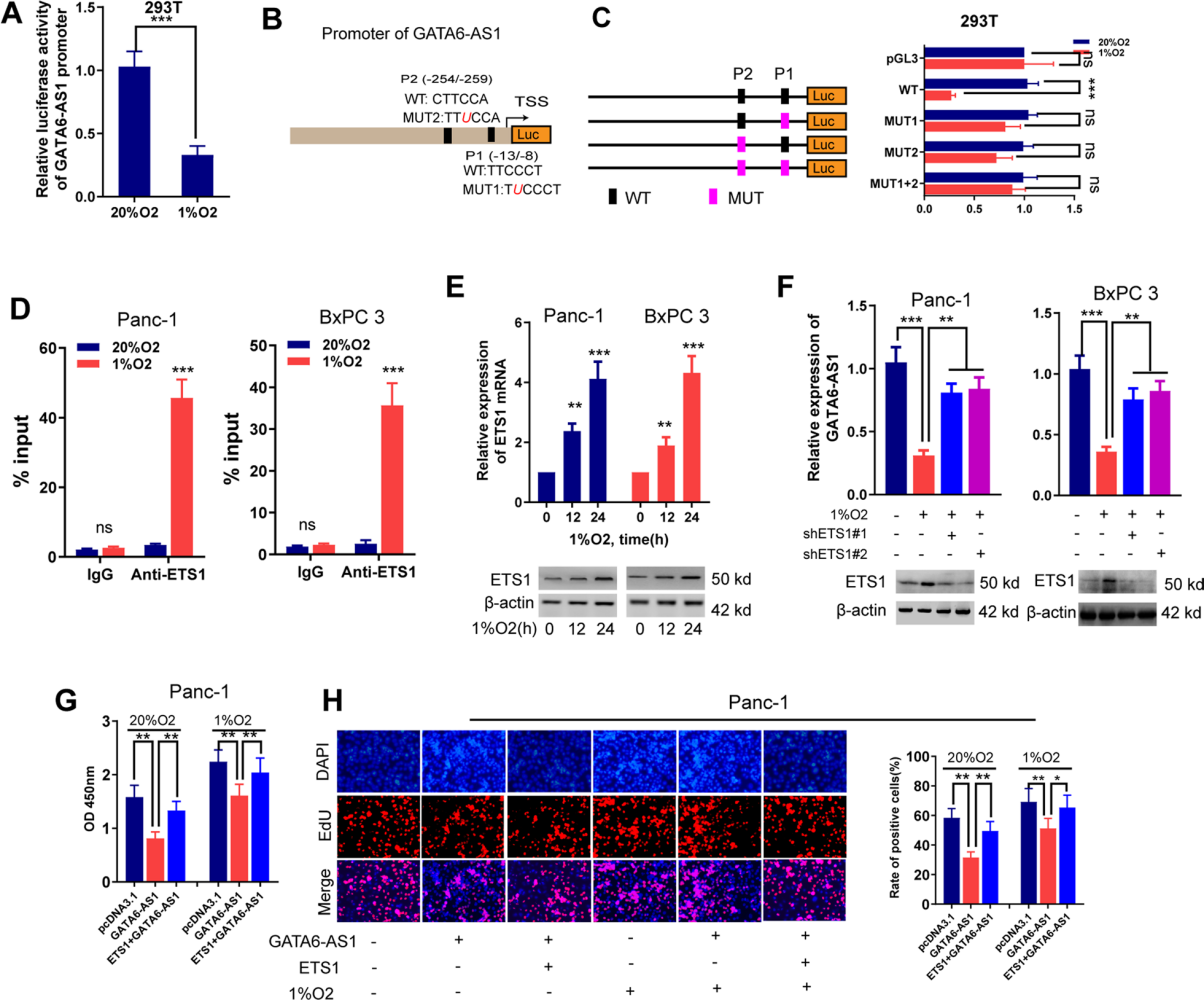
Hypoxia represses GATA6-AS1 expression in PDAC through ETS1 expression at the transcriptional levels. **A** Luciferase activity assay analysis of the reporter constructs containing 2 kb of the GATA6-AS1 promoter in 293T cells exposed to 1% or 20% O_2_. **B** A schematic diagram illustrating the two putative ETS1 binding sites (P1 and P2) in the GATA6-AS1 promoter. C, Luciferase report assays for the GATA6-AS1 promoter region containing either wild-type (WT) or mutated (MUT1, MUT2, MUT1 + 2) ETS1 binding sites in 293T cells exposed to 1% or 20% O_2_. The GATA6-AS1 gene promoter was predicted by using JASPAR (http://jaspar.genereg.net/) online tool. **D** ChIP analysis of ETS1 enrichment at the GATA6-AS1 promoter for lysates from Panc-1 and BxPC 3 cells exposed to 1% or 20% O_2_. ChIP products were amplified by qPCR. IgG was used as controls. E, RT-qPCR and western blot assays analysis of ETS1 expression in Panc-1 and BxPC 3 cells exposed to 1% or 20% O_2_. **F** RT-qPCR assay analysis of GATA6-AS1 expression in Panc-1 and BxPC 3 with transfection of shRNAs targeting ETS1 or shNC under 1% or 20% O_2_ conditions. G-H, MTT (**G**) and Edu (**H**) assays analysis of cell viability in Panc-1 and BxPC 3 cells transfection with the indicated vectors under 1% or 20% O_2_ conditions. *HIF* Hypoxia-inducible factor, *NC* negative control, *ChIP* chromatin immunoprecipitation, *ns* not significant. Data represent mean ± S.D. from three independent experiments. *P < 0.05; **P < 0.01; ***P < 0.001

We then performed experiments to implore the upstream mechanism leading to GATA6-AS1 downregulation under hypoxia conditions. We first carried out a luciferase activity assay for the reporter constructs with 2 kb of the GATA6-AS1 promoter, and the results manifested that incubation of 293T cells under hypoxia (1% O_2_) led to a significant decrease in luciferase activity compared to cells under normoxia (20% O_2_), implying that the mechanism is probably transcriptional (Fig. 3A). As well known, HIF1A and HIF2A are the principal hypoxia-dependent transcriptional factor in modulating hypoxia-linked gene expression. To explore whether HIF1A or HIF2A inhibits the expression of GATA6-AS1, we transfected two parallel shRNAs targeting HIF1A or HIF2A in Panc-1 and BxPC 3 cells under hypoxia, respectively. RT-qPCR, as well as western blotting assays, noticed that shRNAs could substantially inhibit the expression levels of HIF1A and HIF2A, respectively, especially for shHIF1A#2 and shHIF2A#2 (Additional file 1: Fig. S6A, B). We then investigated the impacts of HIF1A or HIF2A on GATA6-AS1 expression levels and found that suppression of HIF1A or HIF2A cannot affect GATA6-AS1 levels in hypoxia-exposed PDAC cells, affirming that hypoxia-suppressed GATA6-AS1 was not dependent on HIF1A or HIF2A (Additional file 1: Fig. S6C, D). To determine the probable modulating transcription factors for hypoxia-inhibited GATA6-AS1, we did a bioinformatics analysis utilizing JASPAR (http://jaspar.genereg.net/) and revealed that the GATA6-AS1 promoter segment had ETS1 attachment sites at remarkable proximity to the transcription initiation site (Fig. 3B). Previous investigations affirmed that ETS1 induced by hypoxia attenuates the transcription of downstream genes once it attaches to portions with remarkable proximity to the transcription starting site [21, 22]. We thus conducted the luciferase activity analysis to examine if ETS1 could attach to the GATA6-AS1 promoter, and the results suggested that mutation on the GATA6-AS1 promoter attenuated the influence of hypoxia on promoter-luciferase activity (Fig. 3C). We also performed ChIP experiments and determined a substantial enrichment in the binding of ETS1 to the promoter portion of GATA6-AS1 in cells under hypoxic environments (Fig. 3D). To examine whether ETS1 expression was hypoxia-responsive, we first examined ETS1 expression under hypoxic conditions utilizing RT-qPCR and western blotting assays, and findings affirmed an increased mRNA and protein levels of ETS1 expression under hypoxia compared with normoxic controls (Fig. 3E). Furthermore, data from TCGA-PAAD showed a significantly positive association between ETS1 and HIF1A or HIF2A (Additional file 1: Fig. S6E). To evaluate whether the downregulated GATA6-AS1 under hypoxia was through ETS1, we knocked down ETS1 expression via two parallel shRNAs targeting ETS1 (shETS1#1 and shETS1#2), and the findings displayed that silencing of ETS1 rescued GATA6-AS1 expression in hypoxia-treated PDAC cells (Fig. 3F). Furthermore, research in human PDAC tissues showed that GATA6-AS1 was negatively correlated with ETS1 in TCGA-PAAD (*R* = − 0.331, P < 0.0001), GSE15471 (*R* = − 0.2712, P = 0.0412) and cohort B (*R *= − 0.3328, P = 0.0043; Additional file 1: Fig. S6F–H). We next examined whether overexpression of ETS1 could restore GATA6-AS1-inhibited tumor aggressive phenotype under hypoxic conditions. Indeed, overexpression of GATA6-AS1 inhibited PDAC cells proliferation, migration, invasion, and EMT, while further overexpression of ETS1 led to a significant rescue in tumor aggressive phenotype in both normoxic and hypoxic settings (Fig. 3G, H and Additional file 1: Fig. S6I–K). In summary, our data demonstrate that hypoxia suppresses GATA6-AS1 via ETS1 expression at the transcriptional levels.

### GATA6-AS1 suppresses EMT under hypoxia by inhibiting SNAI1 mRNA stability

**Fig. 4:**
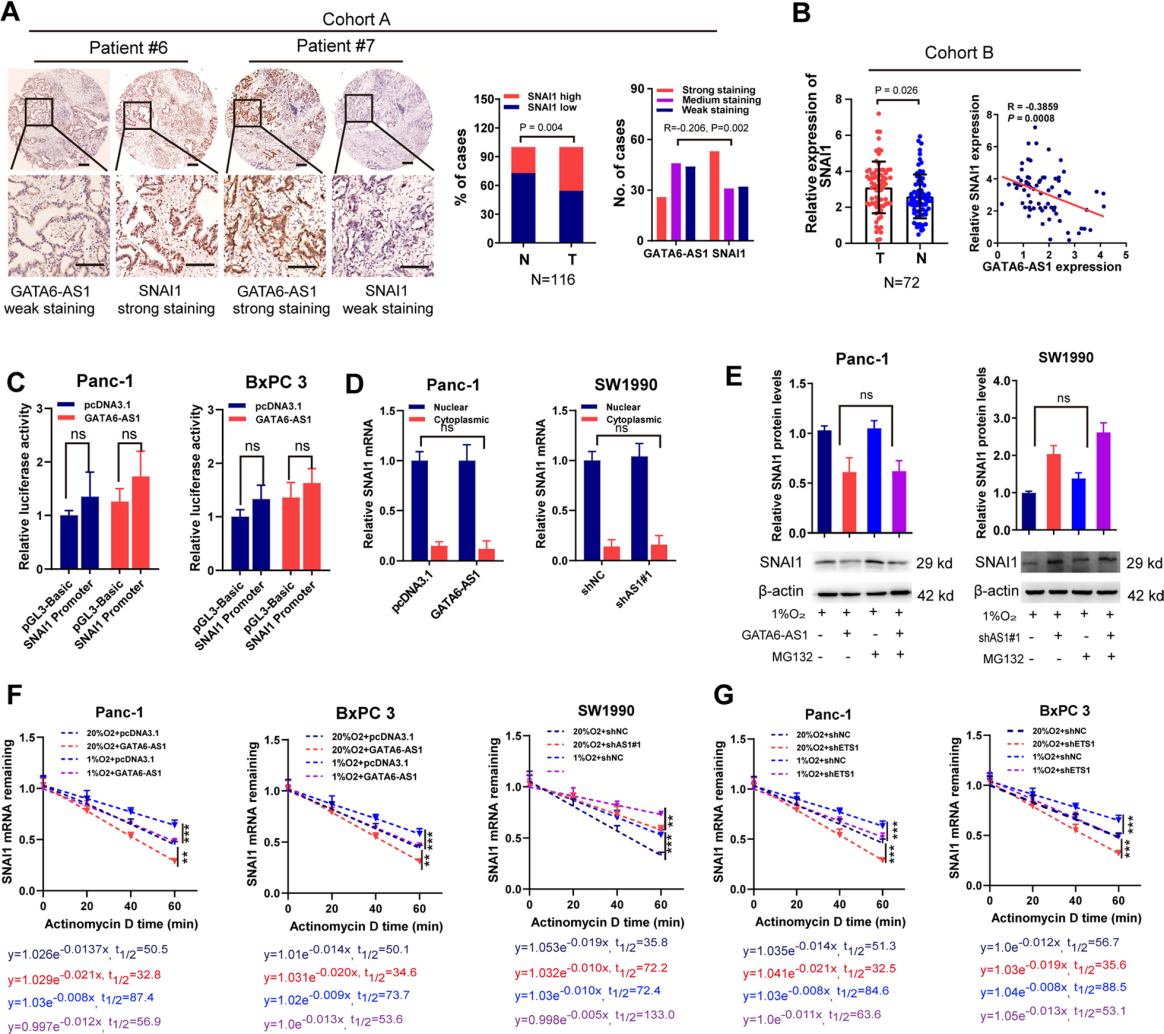
GATA6-AS1 suppresses EMT under hypoxia through inhibiting SNAI1 mRNA stability. **A**, **B** Immunohistochemical staining and RT-qPCR analysis of SNAI1 expression in human PDAC tissues in cohort A and cohort B, respectively. The *R*-values and *P*-values were derived via Spearman correlation analysis. Scale bar, 200 μm. **C** Luciferase reporter assay analysis of SNAI1 promoter activity in Panc-1 and BxPC 3 cells with transfection of GATA6-AS1 overexpression vector or control. **D** Cellular fractionation experiments analysis of SNAI1 RNA subcellular distribution of PDAC cells transfection with the indicated vectors. **E** Western blot assays analysis of SNAI1 protein in PDAC cells with MG132 treatment. PDAC cells were transfected with the indicated vectors and exposed to 1% or 20% O_2_. **F**, **G** RNA stability assay analysis of SNAI1 mRNA in PDAC cells with Actinomycin D treatment. PDAC cells were transfected with the indicated vectors and exposed to 1% or 20% O_2_. Data represent mean ± S.D. from three independent experiments. *P < 0.05; **P < 0.01; ***P < 0.001

Diverse lines of evidence depict that the EMT process is organized by EMT-TFs. As denoted in Fig. 2H, I, overexpression of GATA6-AS1 lowered the mRNA as well as protein expression levels of EMT-TFs. Further analyses clearly indicated that the EMT process was influenced to a higher degree by changes in SNAI1 expression under hypoxia, which implied that SNAI1 accounts for more important roles than others in the involvement of GATA6-AS1 in the EMT process. Moreover, a negative correlation between SNAI1 mRNA and GATA6-AS1 level was found in human PDAC tissues from TCGA-PAAD (*R* = − 0.218, *P* = 0.0034) and GSE15471 (*R* = − 0.3262, *P* = 0.0427; Additional file 1: Fig. S3B, C). To collaborate on our findings, we evaluated SNAI1 expression levels in our two cohorts of PDAC tissues. Results confirmed that higher levels of SNAI1 were discovered in most cancerous tissues, and expression levels of SNAI1 were inversely associated with those of GATA6-AS1 (*R* = − 0.206, *P* = 0.002 for cohort A; *R* = − 0.3859, *P* = 0.008 for cohort B, Fig. 4A, B). These results suggested that hypoxic GATA6-AS1 is closely correlated with SNAI1 expression. Furthermore, we carried out gain and loss-of-function studies and ascertained that SNAI1 was a malignant factor to trigger proliferation, migration, invasion, and EMT in PDAC cells under normoxic and hypoxic conditions (Additional file 1: Fig. S7A–J). We thus focused on SNAI1 for the subsequent experiments.

Luciferase reporter assay explored whether GATA6-AS1 could impact the expression of SNAI1 at the transcriptional level. The data revealed that the overexpression of GATA6-AS1 had no influence on the promoter activity of SNAI1 in Panc-1 as well as BxPC 3 cells (Fig. 4C), indicating that GATA6-AS1 may regulate SNAI1 at the posttranscriptional level. Moreover, there was no difference in subcellular localization of SNAI1 mRNA after changing the GATA6-AS1 expression in PDAC cells (Fig. 4D), suggesting that GATA6-AS1 had no effects on SNAI1 mRNA transport. To determine whether GATA6-AS1 had effects on SNAI1 proteasomal degradation, we used the proteasome inhibitor MG132 to detect the SNAI1 protein level in hypoxic conditions. The data validated that treatment with MG132 did not lead to significant changes in the SNAI1 protein level by manipulating GATA6-AS1 expression under hypoxic conditions (Fig. 4E). These results indicated that the regulation of SNAI1 by GATA6-AS1 was not through modulating the proteasomal degradation. Thus, we suspected whether GATA6-AS1 regulated SNAI1 expression by regulating SNAI1 mRNA stability. To this end, mRNA degradation assays were done utilizing Act D to suppress de novo mRNA transcription. We found that exposure of Panc-1 and BxPC 3 to hypoxia increased the SNAI1 mRNA stability. However, this effect of hypoxia was partially abolished in GATA6-AS1 overexpression or strengthened in GATA6-AS1 knockdown subclones (Fig. 4F). Furthermore, ETS1 silencing prohibited hypoxia-induced stability of SNAI1 mRNA (Fig. 4G). Collectively, these results indicated that hypoxia enhances SNAI1 mRNA stability through the inhibition of GATA6-AS1.

### GATA6-AS1 inhibits SNAI1 mRNA stability via FTO-mediated m6A demethylation manner

**Fig. 5:**
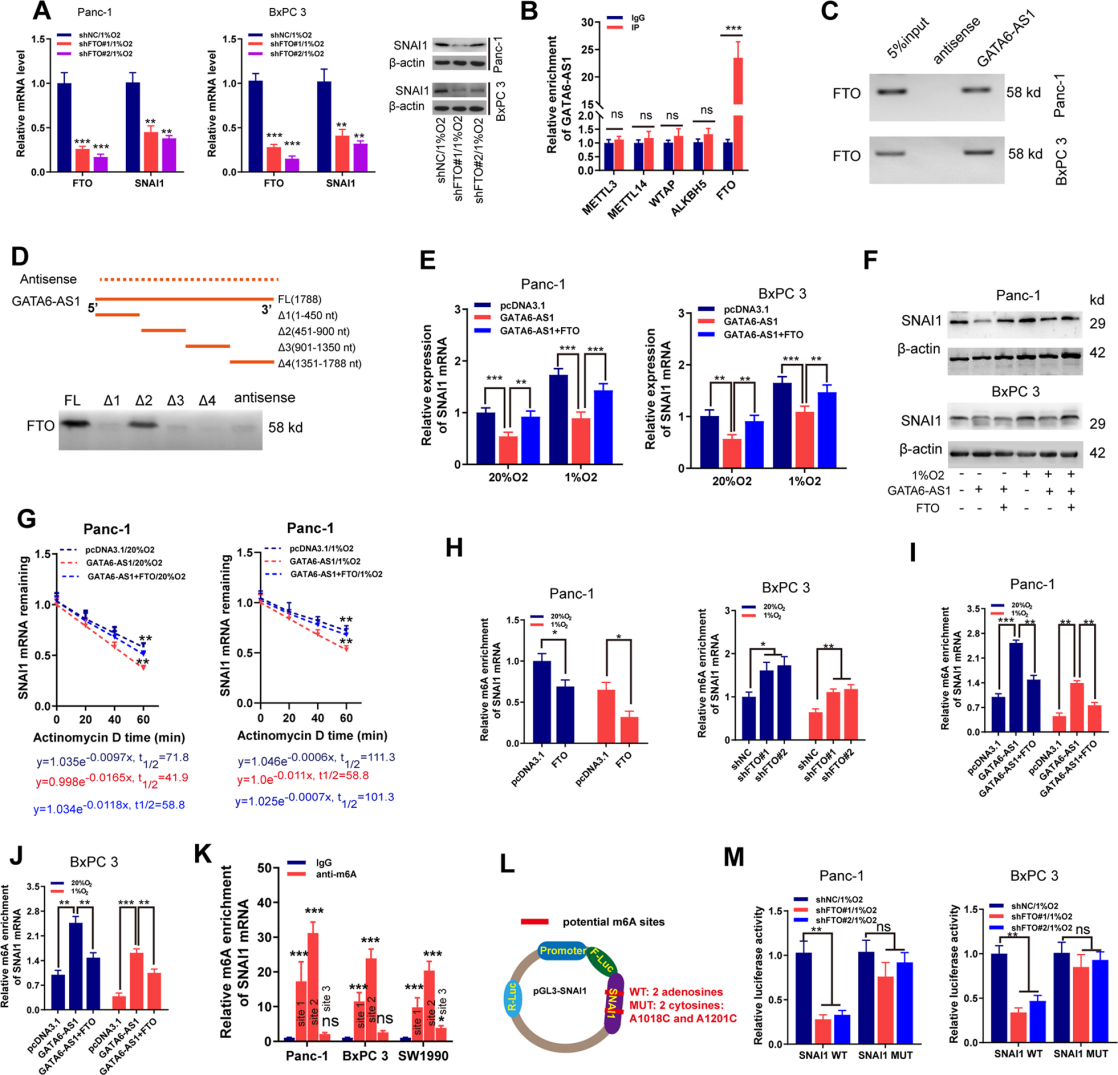
GATA6-AS1 increases SNAI1 mRNAs degradation via repressing FTO expression. **A** RT-qPCR and western blotting analysis of SNAI1 expression in PDAC cells after transfection with the indicated vector under 1% O_2_. **B** RIP assays were performed in Panc-1 cells to search the possible upstream m6A enzymes (METTL3, METTL14, WTAP, ALKBH5 and FTO), which might bind with GATA6-AS1. **C** RNA pull-down and western blot assays were performed to confirm the association between FTO and GATA6-AS1. **D** RNA pull-down using sequentially deleted GATA6-AS1 fragments demonstrates the binding segment of GATA6-AS1 with FTO. **E**, **F** RT-qPCR and western blotting analysis of SNAI1 expression in PDAC cells with transfection of the indicated vector under 1% or 20% O_2_. **G** RNA stability assays analysis of SNAI1 mRNA in PDAC cells transfection with indicated vectors and treatment with Actinomycin D. **H**–**J** Me-RIP and qPCR assay analysis of the relative m6A enrichment of SNAI1 mRNA in PDAC cell lines with transfection of the indicated vectors under 1% or 20% O_2_ conditions. **K** MeRIP combined with RT-qPCR analysis of the relative m6A enrichment at three sites of SNAI1 transcript predicted by the online tools (SRAMP) in PDAC cell lines. **L** The schematic illustration was established for the luciferase reporter. Different from the wild-type group, two adenosines were replaced by cytosines in mutant group. **M** Wild-type or mutant SNAI1 of luciferase reporters were transfected into PDAC cells (with the shRNA vectors or controls), followed by the measurement of luciferase activity. *NC* negative control; Data are shown as the mean ± SD of three independent experiments. *P < 0.05; **P < 0.01; ***P < 0.001

Recently, RNA stability was reported to be modulated by m6A modification [13, 23]. Among the m6A enzymes (such as FTO, WTAP, METTL3, METTL14, and ALKBH5), only the inhibition of FTO could obviously change the expression of SNAI mRNA (Fig. 5A and Additional file 1: Fig S8A–D). Further analysis using the TCGA-PAAD dataset showed that FTO was negatively linked to GATA6-AS1 and positively with EMT markers in PDAC samples (Additional file 1: Fig. S8E). So, we selected FTO as the potential m6A enzyme for further analysis. Because GATA6-AS1 was also located in the nucleus, we first explored whether it acted as the regulator of DNA methylation. Then, the CpG island location of FTO promoter regions was predicted by http://www.urogene.org (Additional file 1: Fig. S8F). We found no effects of GATA6-AS1 overexpression on DNA methylation level on FTO promoter region, as demonstrated by MSP experiments (Additional file 1: Fig. S8G). In addition, overexpression of GATA6-AS1 exerts no effects on the expression of DNMT1, DNMT3A, DNMT3B, all of which played crucial roles in DNA methylation [24] (Additional file 1: Fig.S8H). M6A enzymes commonly functioned as RNA-binding proteins (RBPs) by attaching to respective transcripts. The binding of FTO with GATA6-AS1 was first predicted by using a public bioinformatics resource (http://pridb.gdcb.iastate.edu/RPISeq/results.php; Additional file 1: Fig. S8I). Then, RIP assays were also conducted to determine which m6A regulator could bind to GATA6-AS1, and results demonstrated that GATA6-AS1 was enriched by FTO rather than other enzymes (Fig. 5B). The further RNA pull-down and Western blot assays confirmed the binding between FTO and GATA6-AS1 in Panc-1 and BxPC 3 cells (Fig. 5C). To identify the unique binding sites, we took advantage of a series of deletion mutants of GATA6-AS1 to map the FTO binding region, and results showed GATA6-AS1 mutants Δ2 bound to FTO as efficiently as full-length GATA6-AS1, whereas other mutants completely lost their binding capacity (Fig. 5D), indicating that nucleotides 451–900 of GATA6-AS1 are required for the association with FTO. These results suggested that GATA6-AS1 could directly bind to FTO and inhibit its expression.

We then explored the involvement of FTO in regulating SNAI1 expression. We first used a plasmid system to specifically elevate the intracellular level of FTO in PDAC cells and validated the FTO overexpression efficiencies (Additional file 1: Fig. S9A). RT-qPCR, as well as western blot assay, manifested that FTO expression levels induced by hypoxic conditions were reversed by overexpression of GATA6-AS1 and strengthened by depletion of GATA6-AS1 (Additional file 1: Fig. S9B, C). In addition, GATA6-AS1 overexpression markedly decreased SNAI1 expression, while further co-transfection of FTO overexpression plasmid could partially attenuate these effects in PDAC cells, suggesting that the impact of GATA6-AS1 on SNAI1 expression was mediated by FTO expression (Fig. 5E, F). Furthermore, analysis of human PDAC specimens confirmed the positive association between FTO and SNAI1 in our cohort B and GSE15471 (Additional file 1: Fig. S9D, E). We also performed mRNA degradation and qPCR analysis, and the findings revealed that GATA6-AS1 overexpression significantly suppressed, while force expression of FTO abolished the suppression of SNAI1 mRNA stability under normoxia and hypoxic conditions (Fig. 5G and Additional file 1: Fig. S9F).

As an m6A eraser protein, FTO has been reported to regulate tumor progression by inhibiting the m6A modification of some oncogenes [12, 25]. Hence, we employed MeRIP following qPCR assay and demonstrated that FTO silencing increased m6A levels of SNAI1 mRNA under normoxia and hypoxia, while FTO overexpression exerted the opposite effects (Fig. 5H). In addition, FTO overexpression may reverse the increased m6A levels of SNAI1 mRNA caused by GATA6-AS1 overexpression (Fig. 5I, J). We also analyzed the m6A levels of GATA6-AS1 under hypoxia conditions by MeRIP-qPCR assay, and results revealed that hypoxia did not lead to the changed enrichment levels of m6A on GATA6-AS1 transcripts in PDAC cells (Additional file 1: Fig. S9G). These data indicated that, under hypoxic conditions, GATA6-AS1 and FTO contributed to SNAI1 expression via changing m6A levels of SNAI1 mRNA. To confirm the m6A-guided regulation of the SNAI1 transcript, the possible m6A sites on its sequences were determined utilizing the SRAMP online program. We found 6 potential m6A modification sites on the SNAI1 transcript, among which, 3 loci with very high or high reliable sites on the 3′UTR at 1018 bp (site1), 1201 bp (site2), and 1470 bp (site3) from the 5′-end were shown (Additional file 1: Fig. S9H). Eventually, MeRIP analysis revealed that SNAI1 mRNA in the three PDAC cell lines were enriched with m6A at various levels, with the highest in site 2 and the lowest in site 3 (Fig. 5K). To further reveal the involvement of FTO-mediated SNAI1 regulation, we created luciferase reporter plasmids by incorporating SNAI1 mRNA with wild type (WT) or 2 mutate type (MUT) m6A sites (A1018C and A1201C, Fig. 5L). When FTO was depleted, the luciferase activity of PDAC cells transfected with wild-type plasmids was attenuated, whereas the activity of the mutant group remained unmodified (Fig. 5M). Previous reports have suggested that the m6A reader YTHDF2 participated in driving the mRNA degradation [26, 27]. We thus evaluated the involvement of YTHDF2 in the GATA6-AS1/FTO/SNAI1-mediated phenotype and results demonstrated that silencing YTHDF2 could inhibit PDAC progression and the stability of SNAI1 mRNA, and rescue these phenotypes upon GATA6-AS depletion or FTO overexpression (Additional file 1: Fig. S10A–E), Conclusively, these data imply that FTO stimulates m6A demethylation of SNAI1 mRNA in PDAC cells and enhances its mRNA stability.

### Dysregulation of the GATA6-AS1/SNAI1 axis is responsible for the malignant behaviors elicited by hypoxia in PDAC

**Fig. 6:**
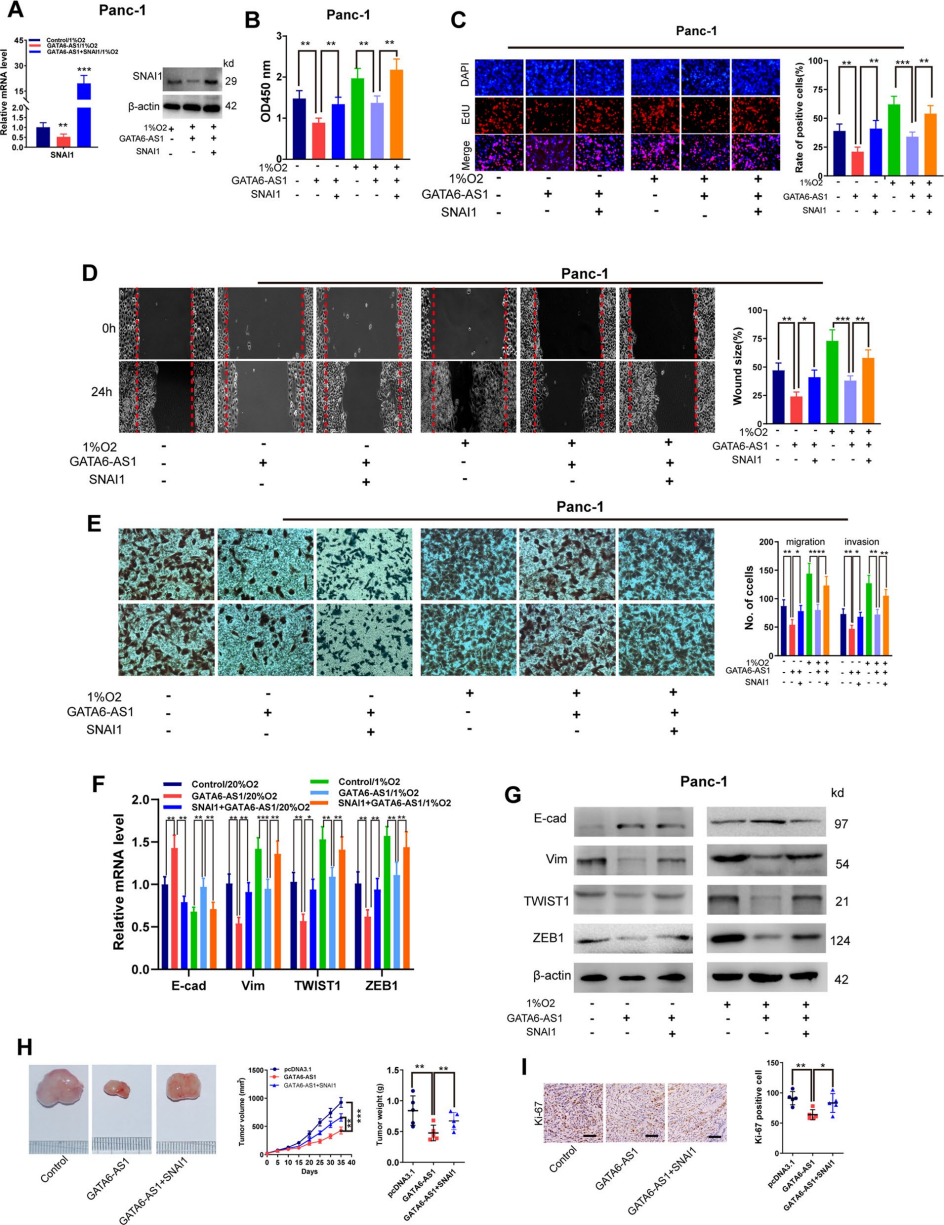
Dysregulation of the GATA6-AS1/SNAI1 axis is responsible for the malignant behaviors elicited by hypoxia in PDAC. **A** RT-qPCR and western blotting analysis of SNAI1 expression in Panc-1 cell lines transfected with the indicated vectors or control under 1% O_2_ conditions. **B**, **C** MTT and Edu assays analysis of cell viability in Panc-1 cells transfection with the indicated vectors under 1% or 20% O_2_ conditions. **D**, **E** The wound healing and transwell assay analysis of cell migration and invasion in Panc-1 cell lines transfected with the indicated vectors under 1% or 20% O_2_ conditions. **F**, **G** RT-qPCR and western blotting analysis of E-cad, Vim, ZEB1 and TWIST1 expression in Panc-1 cells with transfection of the indicated vectors under 1% or 20% O_2_ conditions. **H** Panc-1cell lines transfected with the indicated vectors were injected into the right dorsal of nude mice. Tumor volume curve and tumor weight of subcutaneous xenografts derived from mouse was analyzed. **I** The tumor sections were subjected to immunohistochemistry staining using antibodies against ki-67. Scale bar, 100 μm. Data represent mean ± S.D. from three independent experiments. *P < 0.05; **P < 0.01; ***P < 0.001

To investigate whether GATA6-AS1 restrained malignant behaviors of PDAC cells under hypoxia through modulating the SNAI1 axis, we carried out a series of functional rescue experiments. First, we transfected GATA6-AS1 and SNAI1 overexpression plasmid in Panc-1 cells, and RT-qPCR and western blotting assays demonstrated the construction efficiency (Fig. 6A). MTT detection showed that GATA6-AS1 overexpression prohibited the proliferative effect of PDAC cells under normoxic and hypoxic conditions, while the introduction of SNAI1 almost fully abolished the inhibitory impact of SNAI1 knockdown (Fig. 6B). Comparable results were obtained utilizing EdU assays (Fig. 6C). Wound scratch and transwell assays revealed that overexpression of SNAI1 almost fully reversed the altered capacity of migration and invasion induced by ectopic expression GATA6-AS1 in Panc-1 cells under normoxic and hypoxic conditions (Fig. 6D, E). Concomitantly, the introduction of GATA6-AS1 reduced the mRNA and protein levels of Vim, ZEB1, and TWIST1, but enhanced the expression levels of E-cad, in Panc-1 cells. The further co-transfection of SNAI1 reversed, at least in part, these impacts (Fig. 6F, G). Furthermore, ectopic expression of SNAI1 partially rescued the GATA6-AS1 overexpression-repressed tumor burden and tumor proliferation (Fig. 6H, I). Conclusively, these results deduced that the aberration of the GATA6-AS1/SNAI1 axis may be responsible for tumor progression and EMT process in PDAC cells under normoxic as well as hypoxic conditions.

## Discussion

In the present investigation, we uncovered a novel role of GATA6-AS1 inhibition in EMT process, invasion and metastasis of hypoxic PDAC cells. GATA6-AS1 inhibition may facilitate the stability of SNAI1 mRNA by regulating FTO expression in an m6A-dependent manner. GATA6-AS1 expression is transcriptionally repressed by ETS1 in hypoxic PDAC cells. This modulatory axis is clinically applicable as validated by the analysis of GATA6-AS1/FTO/SNAI1 expression patterns in human PDAC specimens.

Hypoxia can drive tumor cells to adopt aggressive biological behaviors, including proliferative, invasive, and metastatic, as well as EMT progress [28]. Mounting proofs have suggested that many hypoxia-responsive lncRNAs play instrumental roles in tumor malignant phenotypes. Analysis from Wang and his coauthors illustrated that hypoxia-induced lncRNA MAPKAPK5-AS1 contributed to the growth and spread of hepatocellular carcinoma cells through the MAPKAPK5-AS1 and HIF1A signaling loop [29]. Research from Wang et al. affirmed that GATA6-AS1 was a remarkedly downmodulated lncRNA and could repress cell proliferation and invasion via sponging miR-324-5p in lung cancer cells [8]. To our knowledge, the functional roles and mechanisms of GATA6-AS1 in PDAC are yet unknown. In this investigation, we investigated the impact of hypoxia on GATA6-AS1 expression in PDAC. We validated that GATA6-AS1 expression was remarkably downregulated under hypoxia in PDAC cell lines, and low expression of this lncRNA was correlated with poor clinical features and prognosis in PDAC individuals. In vitro as well as in vivo functional experiments have revealed that overexpression of GATA6-AS1 could repress proliferation, invasion, metastasis, and EMT processes, while GATA6-AS1 silencing triggered the contrary effects.

Importantly, the present work noticed that hypoxia in the tumor microenvironment facilitates the downregulation of GATA6-AS1 expression in PDAC cells in a HIF1A or HIF2A-independent manner. We identified ETS1 as the transcriptional inactivator of GATA6-AS1 in PDAC, while the trans-inactivation role of ETS1 in tumors has been previously described [21, 22]. For example, Xing et al. demonstrated that hypoxia facilitates EMT as well as metastasis through ETS1-mediated miR-4521 downregulation [21]. ETS1, an ETS family member of transcription factors, contains the autoinhibitory domain [30]. It has been affirmed that abnormal expression of ETS1 participated in tumor proliferation, metastasis, and EMT in several types of cancer, including PDAC [31]. However, the mechanisms of ETS1 involvement in the hypoxia-induced EMT process have not been fully elucidated in pancreatic cancer cells. Our results revealed that hypoxic conditions induce ETS1 expression, which trans-inactivated GATA6-AS1 in PDAC. ETS1 knockdown abolished GATA6-AS1 inhibition induced by hypoxia. Functionally, overexpression of ETS1 could rescue these tumor-inhibitive phenotypes induced by GATA6-AS1 overexpression under normoxic or hypoxic conditions.

The EMT of tumor cells is a major mechanism of tumor progression [32]. Hypoxic microenvironments induce EMT by regulating EMT-TFs/repressors (such as SNAI1, ZEB1, and TWIST [33]) and activating EMT-associated signaling pathways. Here, we explore whether SNAI1 accounts for the involvement of GATA6-AS1 in hypoxia-induced EMT. Snail is a C2H2 zinc-finger protein, which exerts its effects by decreasing the expression of E-cad by binding to its promoter [34]. SNAI1 was indicated to participate in hypoxia signaling pathways to enhance tumorigenesis [35]. Importantly, using the genetically engineered mouse models (KPC mice) with deletion of SNAI or TWIST, Zheng et al. [36] informed that although suppression of SNAI or TWIST in the primary tumor did not alter the emergence of invasion and blood dissemination of PDAC, it resulted in an increase in gemcitabine sensitivity and overall survival of mice. In our present research, we noticed that hypoxia-induced EMT gene expression could be abrogated by ectopic expression of GATA6-AS1, and be strengthened by GATA6-AS1 depletion. Overexpression of SNAI1 could rescue the inhibitive impacts of GATA6-AS1 overexpression on invasion and EMT of PDAC cells. We further demonstrated that GATA6-AS1 depletion triggered SNAI1 expression via increasing its mRNA stability in PDAC cells.

To explore the underlying mechanisms of SNAI mRNA stability increased by GATA6-AS1 depletion under hypoxic conditions, we examine the involvement of M6A modification of SNAI mRNA, because M6A modification was affirmed to have a principal function in all steps of RNA metabolism [9, 10]. As the m6A “eraser”, FTO is the first evidence of the biological effects of m6A modulatory genes on acute myeloid leukemia (AML) [37]. In that work, FTO was shown to trigger AML cell differentiation by negatively modulating ASB2 and RARA. Likely, FTO has been reported to serve as a key oncogene in glioblastoma [38], esophageal squamous cell carcinoma [23], NSCLC [12], and PDAC [39]. Our research ascertained that FTO was a direct and functional binding partner of GATA6-AS1. It has been reported that FTO can affect mRNA half-life by modulating polyadenylation [40]. Consistent with this, our MeRIP following qPCR assay revealed that GATA6-AS1 overexpression or FTO knockdown increased, while GATA6-AS1 knockdown or FTO overexpression lowered, m6A levels of SNAI1 mRNA under both normoxia and hypoxia. A previous report from Lin et al. found that the m6a “writer” METTL3 promoted SNAI1 translation in liver cancer [41]. Our results showed that METTL3 silencing could reduce m6a levels of SNAI1 mRNA, but not alter SNAI1 mRNA and protein expression. Research from Wang and his coauthors demonstrated that among m6A-altered RNAs, majority of genes (2248/2479 = 90.6%) showed no change in RNA levels [42]. In addition, m6A regulation on mRNA stability could be heterogeneous and be related to target or tumor models [41]. Furthermore, the recognition site between FTO and SNAI1 is validated in the two sites (1018 and 1201 bp from the 5′-end) of SNAI1 mRNA. Recently, several FTO inhibitors have been discovered, although they may not be clinically applicable due to relatively low selectivity and/or low therapeutic efficacy [43]. We also explored the involvement of m6A reader YTHDF2 in the function of GATA6-AS1, and informed that YTHDF2 participated in GATA6-AS1/FTO-mediated phenotype and enhanced the stability of SNAI1 mRNA. In line with our results, Dixit et al. found that YTHDF2 stabilize important oncogenes, such as MYC and VEGF in glioblastoma stem cells in an m6A-dependent manner, suggesting that the precise nature of the interaction between YTHDF2 and target mRNA might be regulated by other unknown factors which might play a role in a cell type-specific manner [44].

**Fig. 7:**
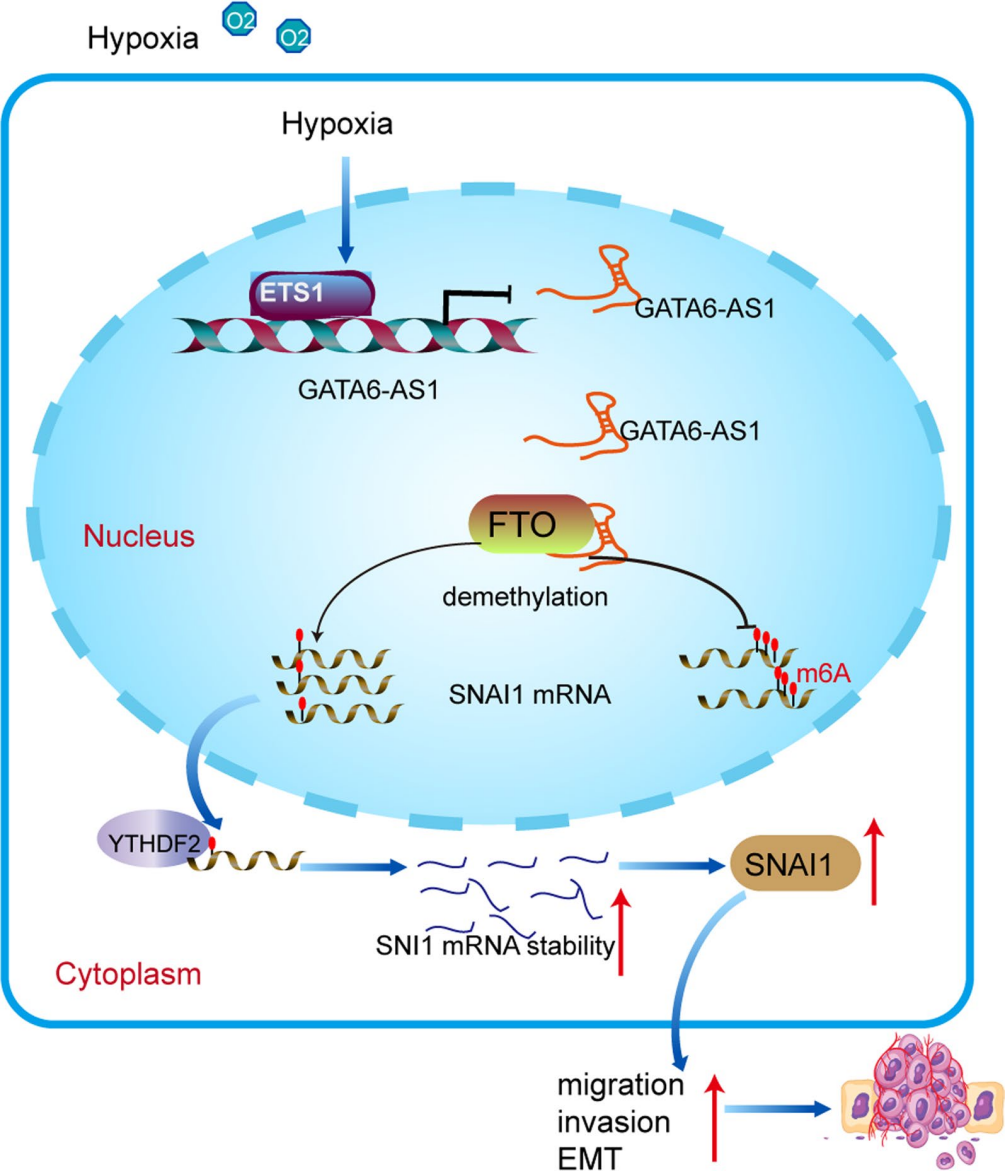
Hypoxia-inhibited GATA6-AS1 suppressed of PDAC cells through the FTO-mediated elevation of SNAI1 mRNA stability

Collectively, targeting lncRNAs will likely play an important role in gene therapy soon, enabling new options for precision medicine [45]. Here, we first demonstrated that hypoxia-inhibited GATA6-AS1 suppressed PDAC cell proliferation, metastasis, and EMT through the FTO-mediated elevation of SNAI1 mRNA stability (Fig. 7), which suggests that GATA6-AS1 might be a potential therapeutic target for refractory hypoxic tumors.

### Supplementary Information

**Additional file 1: Table S1.** shRNA targeting sequence.**Table S2.** The sequences of the primers included in this manuscript. **Figure S1.** Biological characterization of GATA6-AS1. **Figure S2.** GATA6-AS1 overexpression inhibits EMT process in subcutaneous xenografts from mouse. **Figure S3.** Association of GATA6-AS1 expression and epithelial–mesenchymal transition markers in human pancreatic ductal adenocarcinoma tissues. **Figure S4.** GATA6-AS1 knockdown enhances PDAC cells malignant behaviors and EMT.**Figure S5.** GATA6-AS1 knockdown enhances tumor growth and lung metastasis of PDAC cells. **Figure S6.** Hypoxia represses GATA6-AS1 expression in PDAC through ETS1. **Figure S7.** SNAI1 facilitates malignant behaviors and EMT of PDAC cells. **Figure S8.** FTO is positively correlated with EMT markers in the TCGA-PAAD database. **Figure S9.** FTO expression in PDAC. **Figure S10.** YTHDF2 silencing reverses malignant behaviors induced by GATA6-AS1 knockdown in PDAC cells.

## Data Availability

The authors declare that all the data supporting the findings in this study are available in this study and its Additional file, or are available from the corresponding author through reasonable request.
