# Supplementary material for: GATA6-AS1 suppresses epithelial–mesenchymal transition of pancreatic cancer under hypoxia through regulating SNAI1 mRNA stability
Source: J Transl Med. 2023 Dec 6;21:882. doi: 10.1186/s12967-023-04757-5 (PMC10698911; doi:10.1186/s12967-023-04757-5)

**GATA6-AS1 suppresses epithelial-mesenchymal transition of pancreatic cancer through regulating SNAI1 stability**

**Table S1:** shRNA targeting sequence

| Negative control | GAAUACGUACCCCAUUAUA |
| --- | --- |
| GATA6-AS1#1 | ATAAAAGTGACTAAATTGG |
| GATA6-AS1#2 | AAAACAAAAAACCAGAGCC |
| GATA6-AS1#3 | ACAACAACATTCACTACCC |
| SNAI1#1 | TATGGAGAGGAAGAGGGAG |
| SNAI1#2 | TTTGAAATATAAATACCAG |
| HIF1A#1 | CGGCGAAGTAAAGAATCTGAA |
| HIF1A# 2 | TGATGAAAGAATTACCGAATT |
| FTO#1 | TCACCAAGGAGACTGCTATTT |
| FTO#2 | CCCATTAGGTGCCCATATTTA |
| ETS1#1 | GGGACCCGAGCGCGCTCACTT |
| ETS1#2 | GGACCCGAGCGCGCTCACTTC |
| HIF2A#1 | GCCGTACTGTCAACCTCAAGT |
| HIF2A#2 | GCAGCTGCTCCACGCCCAATA |
| ALKBH5#1 | ACAAGUACUUCUUCGGCGA |
| ALKBH5#2 | GCGCCGUCAUCAACGACUA |
| METTL3#1 | | CCUGCAAGUAUGUUCACUATT |  | | --- | --- | |
| METTL3#2 | CGTCAGTATCTTGGGCAAGTT |
| METTL14#1 | CCGGCCATGTACTTACAAGCCGATACTCGAGTATCGGCTTGTAAGTACATGGTTTTT |
| METTL14#2 | CCGGGCTAATGTTGACATTGACTTACTCGAGTAAGTCAATGTCAACATTAGCTTTTT |
| WTAP#1 | GGCAAGTACACAGATCTTAAC |
| WTAP#2 | GCAACACAACCGAAGATGACT |
| YTHDF2#1 | GACTTCTCACACTATGAGAAA |
| YTHDF2#2 | CGGTCCATTAATAACTATAAC |

**Table S2:** The sequences of the primers included in this manuscript

|  | Forward premier (5’-3’) | | Reverse premier (5’-3’) |
| --- | --- | --- | --- |
| **Primers for qRT-PCR** | | | |
| ZEB | ATGATGAATGCGAGTCAGATGC | | ACAGCAGTGTCTTGTTGTTGT |
| E-cadherin | CGAGAGCTACACGTTCACGG | | GGGTGTCGAGGGAAAAATAGG |
| Vimentin | TGCCGTTGAAGCTGCTAACTA | | CCAGAGGGAGTGAATCCAGATTA |
| β-actin | GATCATTGCTCCTCCTGAGC | | ACTCCTGCTTGCTGATCCAC |
| TWIST1 | CGGCCAGGTACATCGACTTC | | CAGAGGTGTGAGGATGGTGC |
| SNAI1 | CTCGGACCTTCTCCCGAATG | | AAAGTCCTGTGGGGCTGATG |
| HIF2A | GACTCCTTCCGACTCCCAGC | | TGGGCCAGCTCATAGAACAC |
| HIF1A | CGTGTTATCTGTCGCTTTGAGTC | | GTCTGGCTGCTGTAATAATGTTCC |
| FTO | TCACCAAGGAGACTGCTATTT | | TCACCAAGGAGACTGCTATTT |
| METTL3 | TTGTCTCCAACCTTCCGTAGT | | CCAGATCAGAGAGGTGGTGTAG |
| METTL14 | AGTGCCGACAGCATTGGTG | | GGAGCAGAGGTATCATAGGAAGC |
| WTAP | CTTCCCAAGAAGGTTCGATTGA | | TCAGACTCTCTTAGGCCAGTTAC |
| ETS1 | TTCCCCTCCCCGGATATGGA | | CCCCGAGTTTACCACGACTG |
| GATA6-AS1 | ATGCGCTTTTTGCCCTGAAG | | AAAGTTGGAGAGCGTCCTCG |
| AC007349.2 | GGGCAAATCACTGCCACTTG | | CGATATTGCTGTGCTGGTGC |
| AC024451.4 | TAAAGTCGCCAGGTCAACCA | | AAAACAGTAGCCCACAGGCA |
| AC091212.1 | TGACAGAAGGCTGCTCTGAA | | TGGCTAATGGGAGACACTGTT |
| AC092053.3 | ACCCATGACCTGACTCCAGA | | CCTCTTGGAGCCTGACTGTG |
| AC245884.10 | GCCATTTGACTTTACGGTATGTT | | TGGGGAGGGAGGAGTTTAAG |
| AL603839.2 | ATGTCCAGGATTTACCAGCTCA | | GGCAGGGGTAGAGTTTCTGT |
| LINC00184 | ATGTCCAGGATTTACCAGCTCA | | GGCAGGGGTAGAGTTTCTGT |
| NAALADL2-AS2 | AGTGACAGTTGCCTTCCTCT | | CTGGCAGTCCAGCATCGTT |
| NUTM2B-AS1 | GCGTCTGGGCCAGGATAAAA | | AGGGCTGGAACACCAGTAAAG |
| ALKBH5 | CGG CGA AGG CTA CAC TTA CG | | CCA CCA GCT TTT GGA TCA CCA |
| YTHDF2 | TAGTCGAAACCTCGTGGTGC | | GGTGGCTGGAGGCAAACTAT |
| DNMT1 | CGTGGTGGTGGATGACAAGA | | CTTCCACGCAGGAGCAGAC |
| DNMT3A | CGGCCATACGGTGGAGCC | | CAGACCTTTAGCCACGACCC |
| DNMT3B | CCGCTTCCTCGCAGCAG | | TGGGCTTTCTGAACGAGTCC |
| **Primers for GATA6-AS1 promoter constructs** | | | |
| GATA6-AS1 P1 | GGTAAGTTCGGGCTGCGATA | | TTTGAGAGCCGCAAGCTTCT |
| GATA6-AS1 P2 | GGGGGCCGTTTTAGGATACC | | GGGGGCCGTTTTAGGATACC |
| **Primers for SNAI1 m6A sites** | | | |
| SNAI1 site1 | AAGATGCACATCCGAAGCCA | | TCATCAAAGTCCTGTGGGGC |
| SNAI1 site2 | CTGTCTGCGTGGGTTTTTGTA | | TCTGTCAGCCTTTGTCCTGT |
| SNAI1 site3 | AGAGTCTGAGATGCCCCGAG | | CCCGACAAGTGACAGCCATT |
| **Primers for MSP** |  | |  |
| FTO, methylation | ATAGTTTTAGACGGGAGTAGGACG | | GAAAACCCGACATACCTTAACTTC |
| FTO, unmethylation | ATAGTTTTAGATGGGAGTAGGATG | | CAAAAACCCAACATACCTTAACTTC |
|  | |  | |

**Additional Figure legends**

**Fig. S1-**Biological characterization of GATA6-AS1

A, RT-qPCR assay analysis of GATA6-AS1 expression in PDAC cells and HPNE cell line. B, GATA6-AS1 locus is located on chromosome 18, next to *GATA6. C,* RT-qPCR assay analysis of GATA6 expression in SW1990 cells response to hypoxia. D-E, **RT-qPCR** and western blot assay of expression levels of GATA6 in SW1990 cells exposed to 1% O_2_ at varying time intervals (0, 12, and 24 h) and concentrations of oxygen (1%, 5% or 20%). F, online analyses of correlation between GATA6-AS1 and GATA6 expression in PDAC cell lines and tissues from CCLE (<https://portals.broadinstitute.org/ccle>) and TCGA dataset. G-H, Cellular fractionation experiments (G) and LncRNA FISH (H)assay of GATA6-AS1 subcellular distribution. I, the protein-coding capacity of GATA6-AS1 was evaluated by LNCipedia ([https://lncipedia.org](https://lncipedia.org/)). J, the secondary structure of GATA6-AS1 is predicted by RNAfold Webserver (http://rna.tbi.univie.ac.at). Data represent mean ± S.D. from three independent experiments. **P* < 0.05; ***P* < 0.01; ****P* < 0.001.


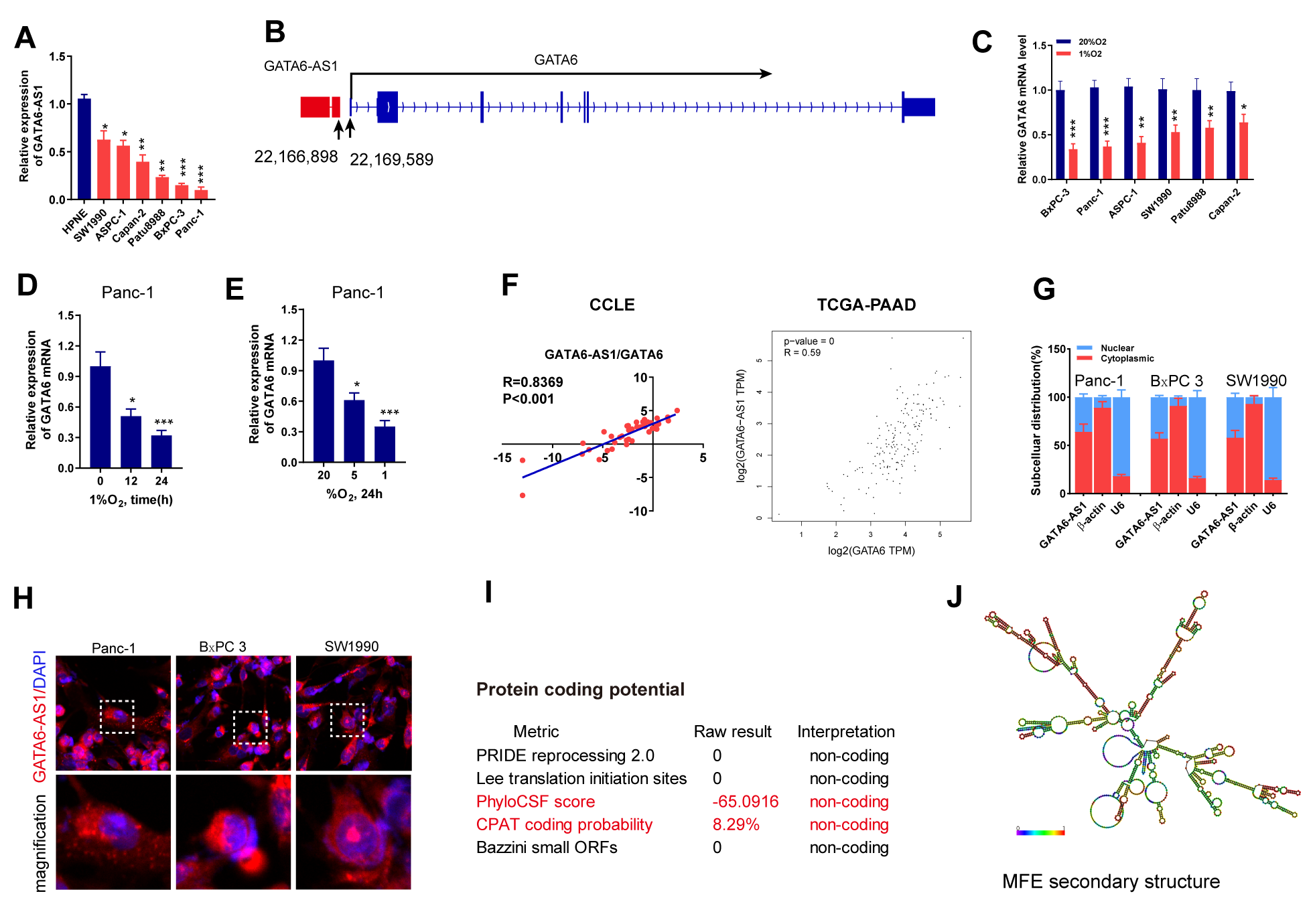


**Fig. S2**-GATA6-AS1 overexpression inhibits EMT process in subcutaneous xenografts from mouse.

A, RT-qPCR assays analysis of EMT markers of Subcutaneous xenografts derived from mouse in PDAC cell lines transfected with GATA6-AS1 or pcDNA3.1. B, IF assays analysis of E-cad and Vim expression in xenografts derived from overexpressing GATA6-AS1 compared with each control. E-cad, E-cadherin; Vim, Vimentin. Data represent mean ± S.D. from three independent experiments. **P* < 0.05; ***P* < 0.01; ****P* < 0.001.


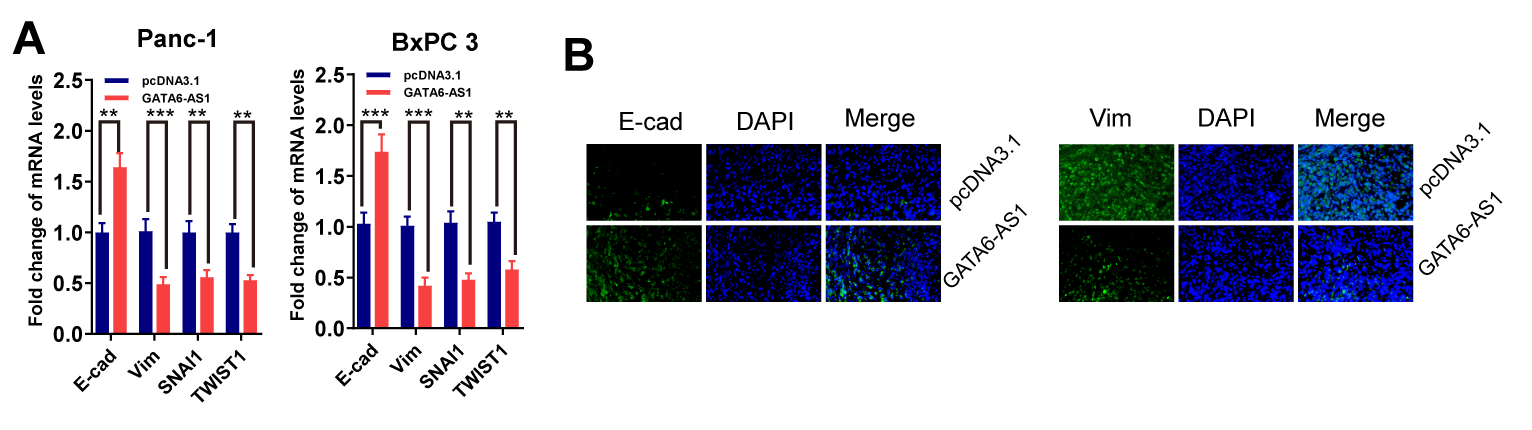


**Fig. S3-** Association of GATA6-AS1 expression and epithelial-mesenchymal transition markers in human pancreatic ductal adenocarcinoma tissues.

A, Immunohistochemical/ISH staining scoring of GATA6-AS1, E-cad and Vim expression levels in cohort A of human PDAC tissue samples (n=116). Scale bar, 200 µm. B-C, Association of GATA6-AS11 expression and epithelial-mesenchymal transition markers in pancreatic ductal adenocarcinoma tissues from TCGA-PAAD dataset (B) and GSE15471 (**C**).


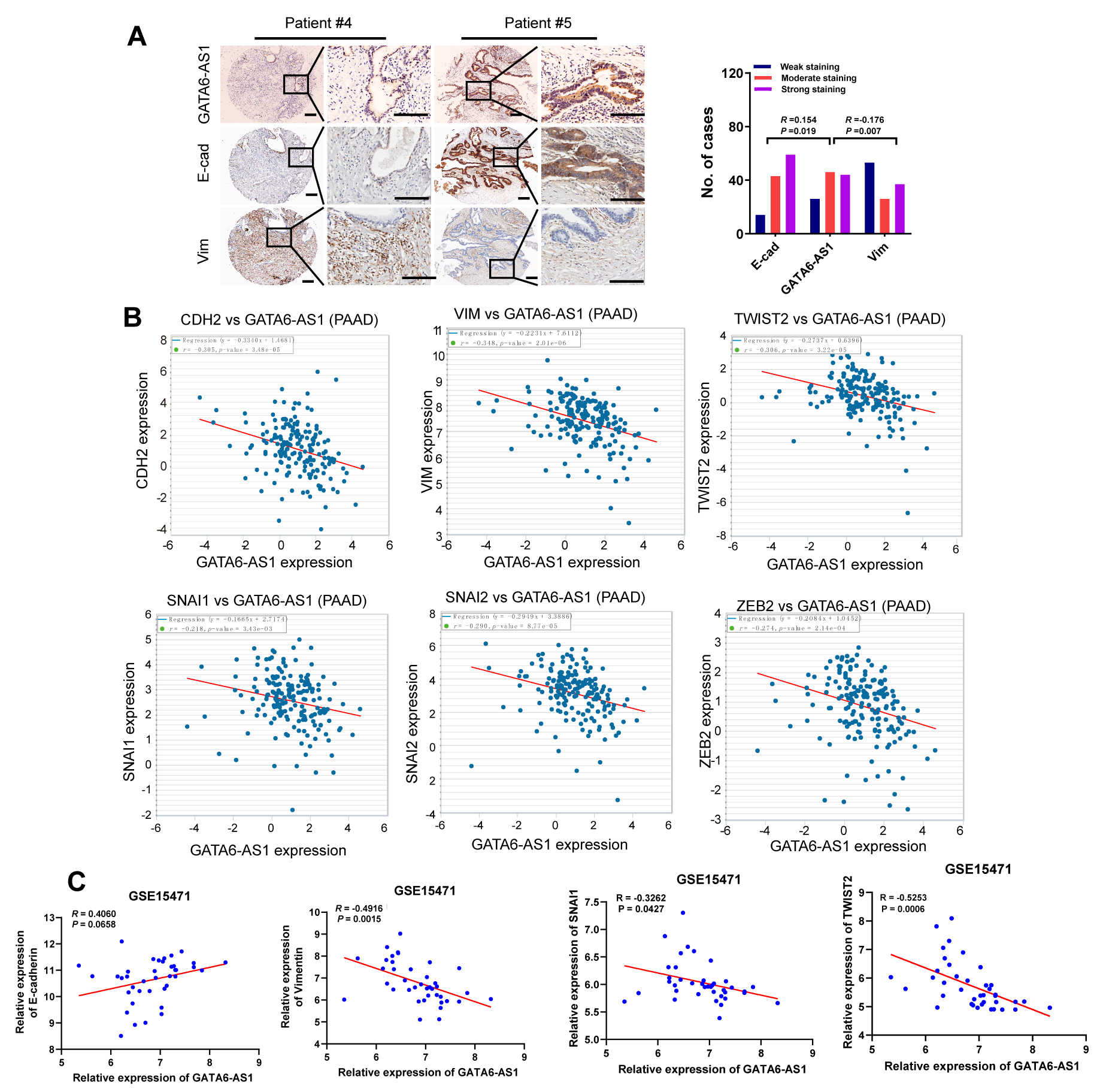


**Fig. S4**- GATA6-AS1 knockdown enhances PDAC cells malignant behaviors and EMT.

A, RT-qPCR assay analysis of GATA6-AS1 expression in PDAC cells after transfection with GATA6-AS1 shRNA vectors and control. B-C, MTT (B) and Edu (C) assays were used to assess cell viability in SW1990 cells transfection with GATA6-AS1 shRNA vectors and control under 1% or 20% O_2_ conditions. D-E, Wound healing and transwell assay analysis of cell migration and invasion in SW1990 cells transfected with GATA6-AS1 shRNA vectors and control under 1% or 20% O_2_ conditions. F, qRT-PCR assays of E-cad, Vim, SNAI1, and TWIST1 expression in SW1990 cells with transfection of GATA6-AS1 shRNA vectors and control under 1% or 20% O_2_ conditions. NC, negative control. Data represent mean ± S.D. from three independent experiments.  *P < 0.05; **P < 0.01; ***P < 0.001.


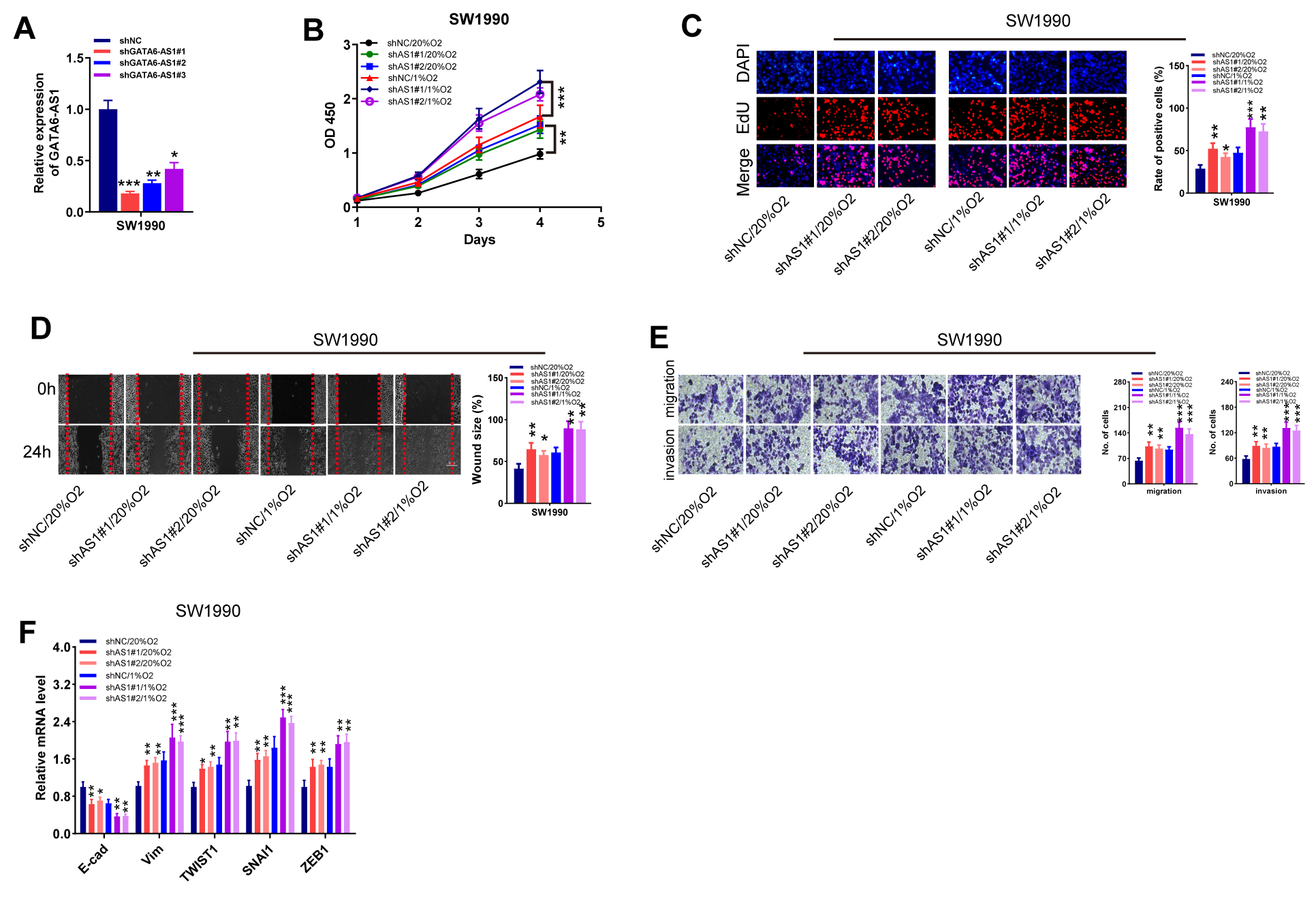


**Fig. S5**- GATA6-AS1 knockdown enhances tumor growth and lung metastasis of PDAC cells

A-B, GATA6-AS1 shRNA-transfected SW1990 cell lines (5× 10^6^ per mouse) were injected into the right dorsal of nude mice. Tumor volume (A) and tumor weight (B) of subcutaneous xenografts derived from mouse was analyzed. C, The tumor sections were subjected to immunohistochemistry staining using antibodies against ki-67. D-F, RT-qPCR, western blot and IHC assays analysis of EMT markers of Subcutaneous xenografts derived from mouse in PDAC cell lines transfected with GATA6-AS1 shRNA vectors and control. G, Representative images of H&E staining of lung sections after injection of SW1990 cells with shNC, shAS#1, and shAS1#2 groups into tail veins of mice (n = 5). Scale bar, 100μm. NC, negative control. Data represent mean ± S.D. from three independent experiments.  *P < 0.05; **P < 0.01; ***P < 0.001.


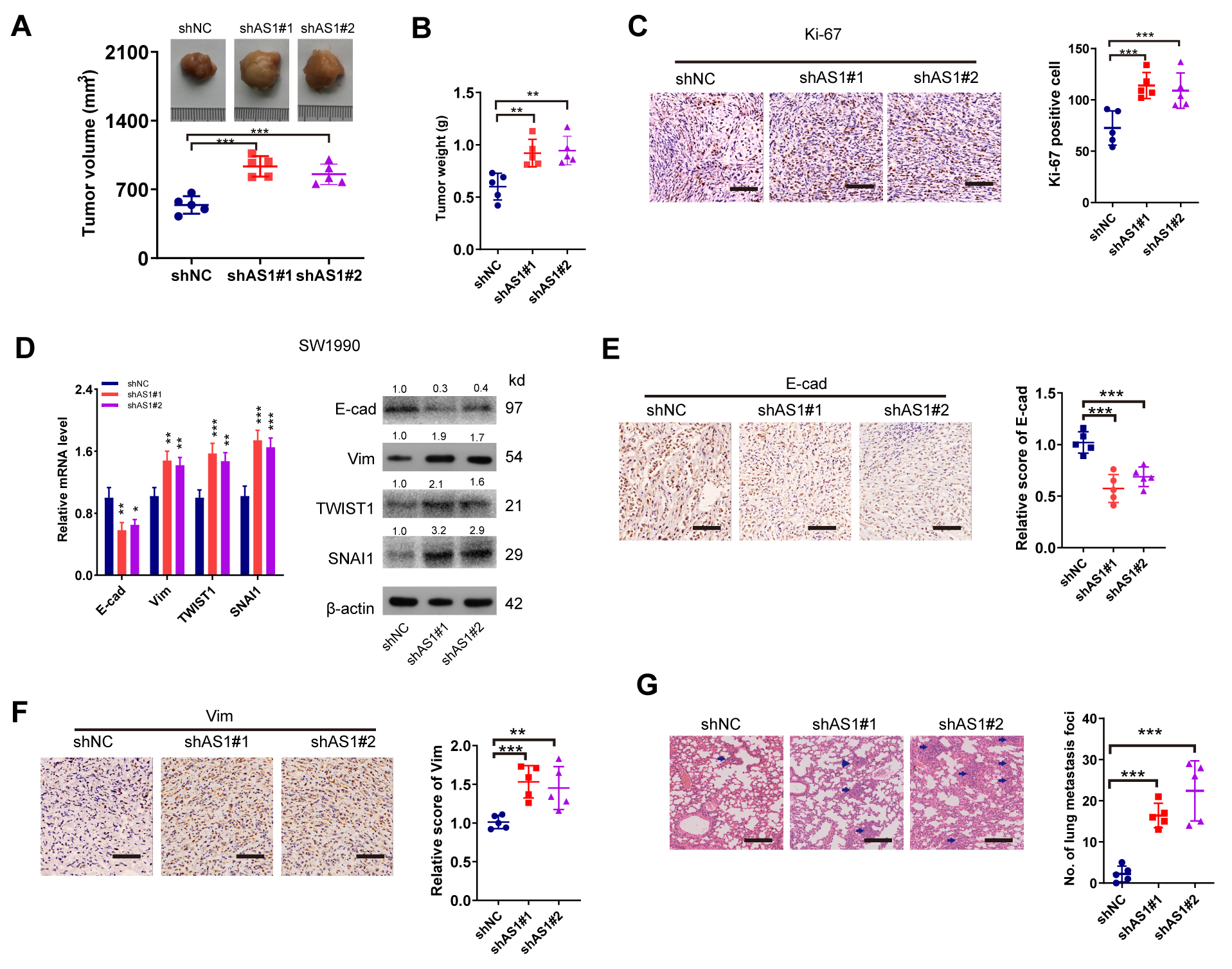


**Fig. S6-** Hypoxia represses GATA6-AS1 expression in PDAC through ETS1.

A-B, RT-qPCR and western blotting assays analysis of the expression levels of HIF1A and HIF2A in Panc-1 and BxPC 3 cells after transfection with the indicated vectors under hypoxia. C-D, RT-qPCR assays analysis of the expression levels of GATA6-AS1 in Panc-1 and BxPC 3 cells after transfection with the indicated vectors under normoxia and hypoxia. E, Association of GATA6-AS1 expression and HIF1A and HIF2A in human tissues from TCGA-PAAD dataset. F-H, Association of GATA6-AS1 expression and ETS1 in pancreatic ductal adenocarcinoma tissues from TCGA-PAAD dataset (F)**,** GSE15471 (G) and cohort B (H). I, The transwell assay analysis of cell migration and invasion in Panc-1 cell lines transfected with the indicated vectors under normoxia and hypoxia. J-K, RT-qPCR and western blot assays of E-cad, Vim, SNAI1, and TWIST1 expression in Panc-1 cell lines transfected with the indicated vectors under normoxia and hypoxia. HIF, Hypoxia-inducible factor; E-cad, E-cadherin; Vim, Vimentin; NC, negative control. Data represent mean ± S.D. from three independent experiments. **P* < 0.05; ***P* < 0.01; ****P* < 0.001.


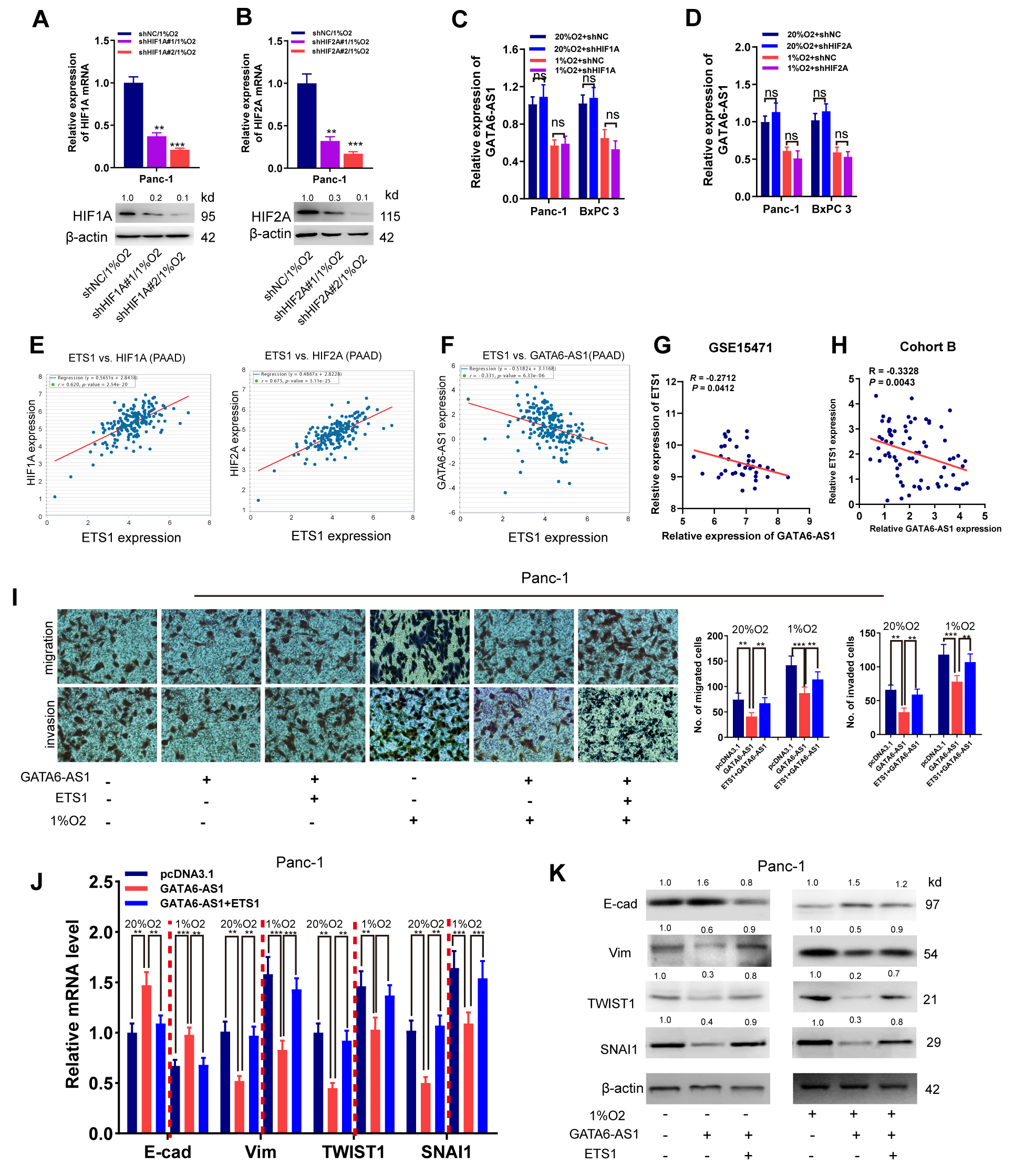


**Fig. S7-** SNAI1 facilitates malignant behaviors and EMT of PDAC cells.

A-D, MTT (A-B) and Edu (C-D) assays were used to assess cell viability in Panc-1 and BxPC 3 cells transfection with SNAI1 overexpression or shRNA vectors and control under 1% or 20% O_2_ conditions. E-H, Wound healing (E-F) and transwell (G-H) assay analysis of cell migration and invasion in Panc-1 and BxPC 3 cells transfection with SNAI1 overexpression or shRNA vectors and control under 1% or 20% O_2_ conditions. I-J, qRT-PCR assays of E-cad, Vim, ZEB1 and TWIST1 expression in Panc-1 and BxPC 3 cells transfection with SNAI1 overexpression or shRNA vectors and control under 1% or 20% O_2_ conditions. Data represent mean ± S.D. from three independent experiments. **P* < 0.05; ***P* < 0.01; ****P* < 0.001.


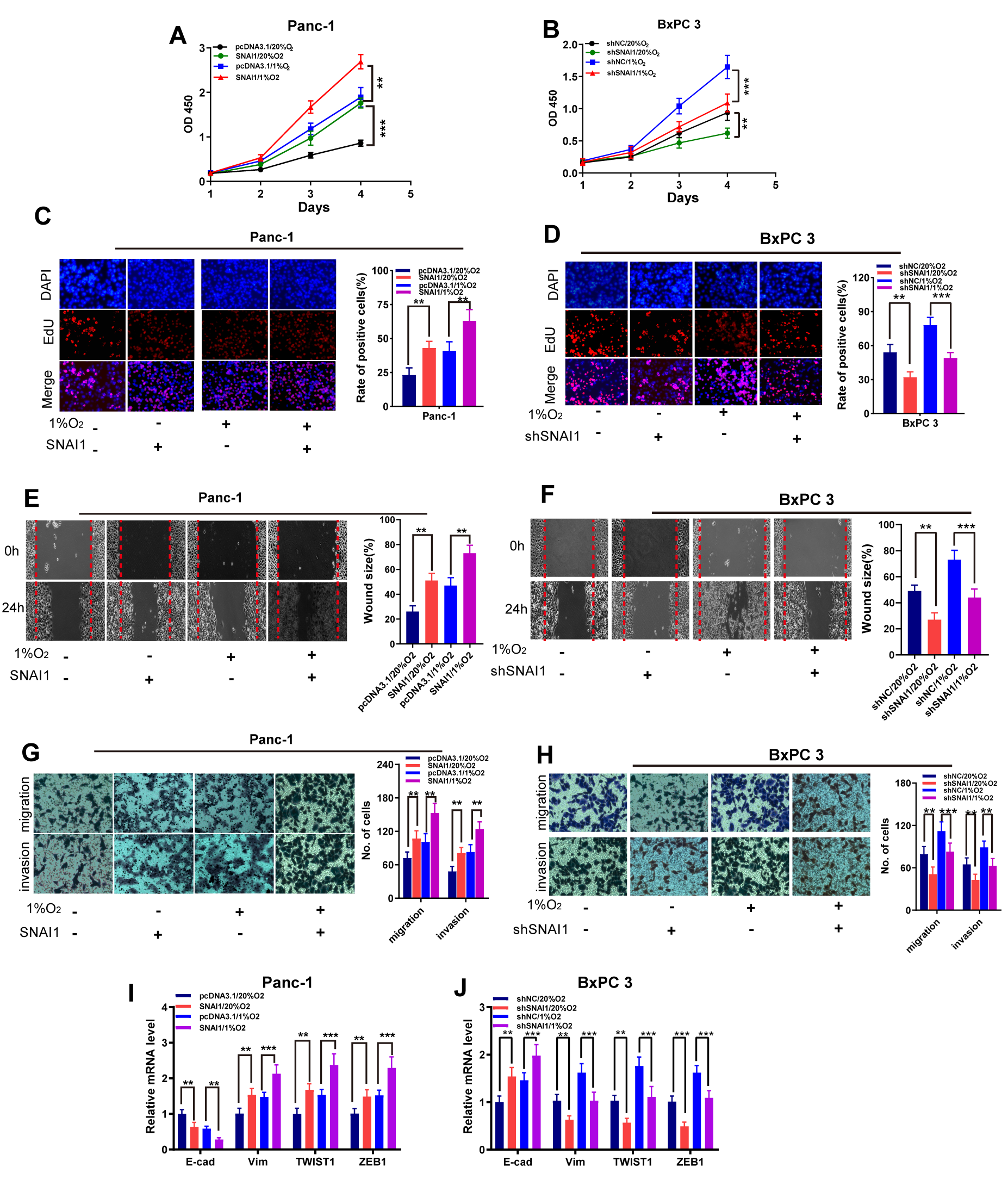


**Fig. S8-** FTO is positively correlated with EMT markers in the TCGA-PAAD database

A-D, RT-qPCR assays analysis of the expression levels of SNAI1 mRNA in Panc-1 cells after transfection with shRNA vectors targeting METTL3, METTL14, WTAP, and ALKBH5 and control vectors. E, FTO is negatively correlated with GATA6-AS1 expression and positively with EMT markers in the TCGA-PAAD database. F, The CpG island location of FTO promoter regions predicted by <http://www.urogene.org/>. G, MSP assays of FTO methylation level in PDAC cells after transfection with GATA6-AS1 overexpression and control vectors. H, RT-qPCR assays analysis of the expression levels of DNMT1, DNMT3a, DNMT3b mRNA in PDAC cells after transfection with GATA6-AS1 overexpression and control vector. I, The probability of the interaction between GATA6-AS1 and FTO was predicted by RNA-Protein interaction prediction (RPISeq: <http://pridb.gdcb.iastate.edu/RPISeq/results.php>). MSP, Methylation-Specific Polymerase Chain Reaction; SVM, Support Vector Machine; RF, Random Forest. Data represent mean ± S.D. from three independent experiments. **P* < 0.05; ***P* < 0.01; ****P* < 0.001.


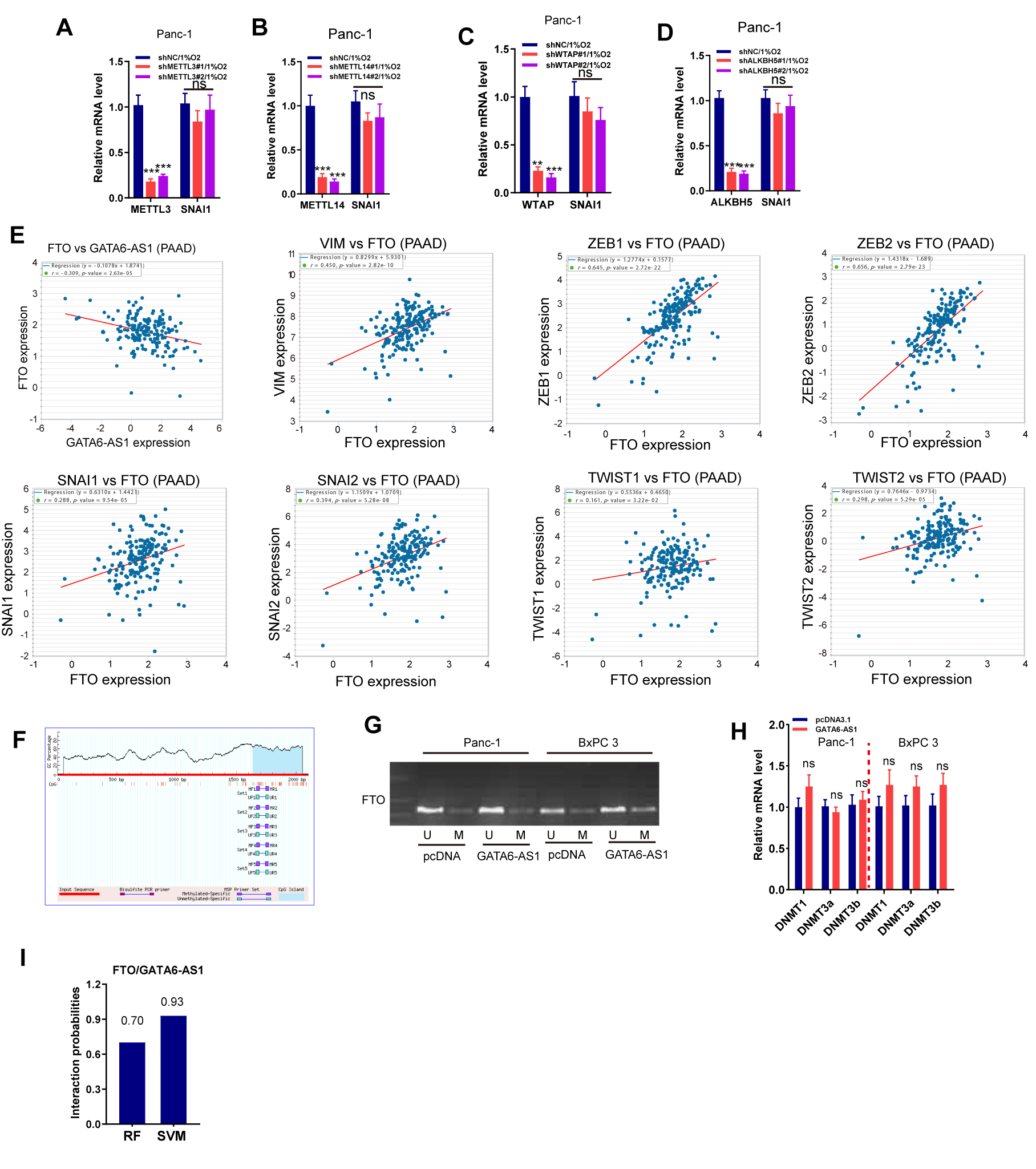


***Fig. S9****-FTO expression in PDAC*

A, RT-qPCR and western blotting assays analysis of the expression levels of FTO in Panc-1 and BxPC 3 cells after transfection with overexpression FTO vectors and control vectors. B-C, RT-qPCR and western blot assay of FTO expression levels in PDAC cells after transfection with the indicated vectors under hypoxia. D-E, Association between FTO and SNAI1 expression in PDAC tissues based on dataset from cohort B and GSE15471. F, GATA6-AS1 overexpression significantly suppressed, while force expression of FTO abolished the suppression of SNAI1 mRNA stability under normoxia and hypoxic conditions. G, MeRIP- qPCR assay of m6A on GATA6-AS1 transcripts in PDAC cells under hypoxia. H, Using the online tools (SRAMP), three potential m6A modification sites with high reliable on the SNAI1 transcript were shown. Data represent mean ± S.D. from three independent experiments. **P* < 0.05; ***P* < 0.01; ****P* < 0.001.


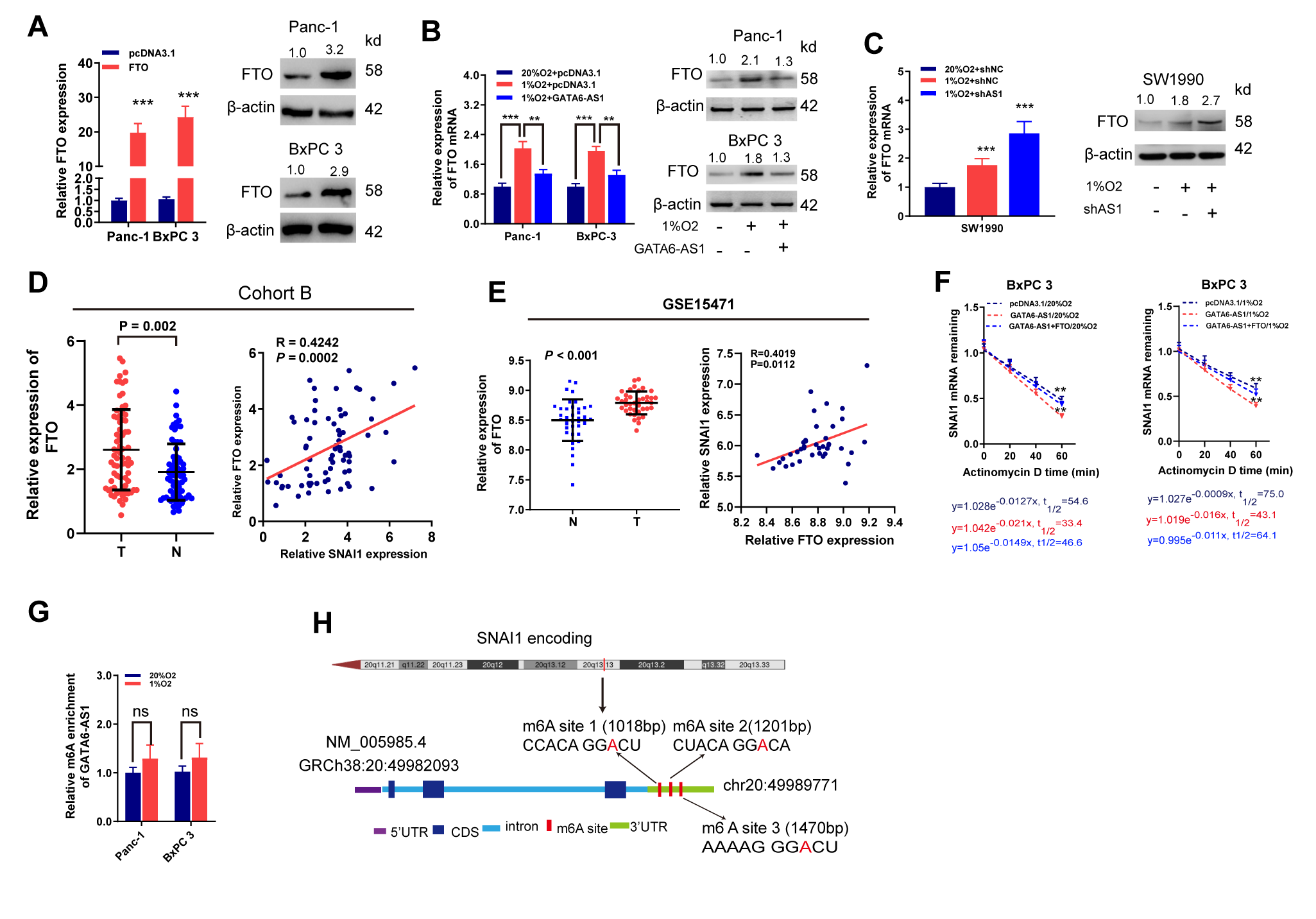


**Fig. S10-** YTHDF2 silencing reverses malignant behaviors induced by GATA6-AS1 knockdown in PDAC cells.

A, RT-qPCR assay analysis of YTHDF2 expression in SW1990 cells after transfection with YTHDF2 shRNA vectors and control. B-C, MTT (B) and Edu (C) assays were used to assess cell viability in SW1990 cells transfection with the indicated vectors under 1% O_2_ conditions. D, Transwell assay analysis of cell migration and invasion in SW1990 cells transfection with the indicated vectors under 1% O_2_ conditions. E, RNA stability assay analysis of SNAI1 mRNA in SW1990 cells transfection with the indicated vectors after Actinomycin D treatment. Data represent mean ± S.D. from three independent experiments. **P* < 0.05; ***P* < 0.01; ****P* < 0.001.


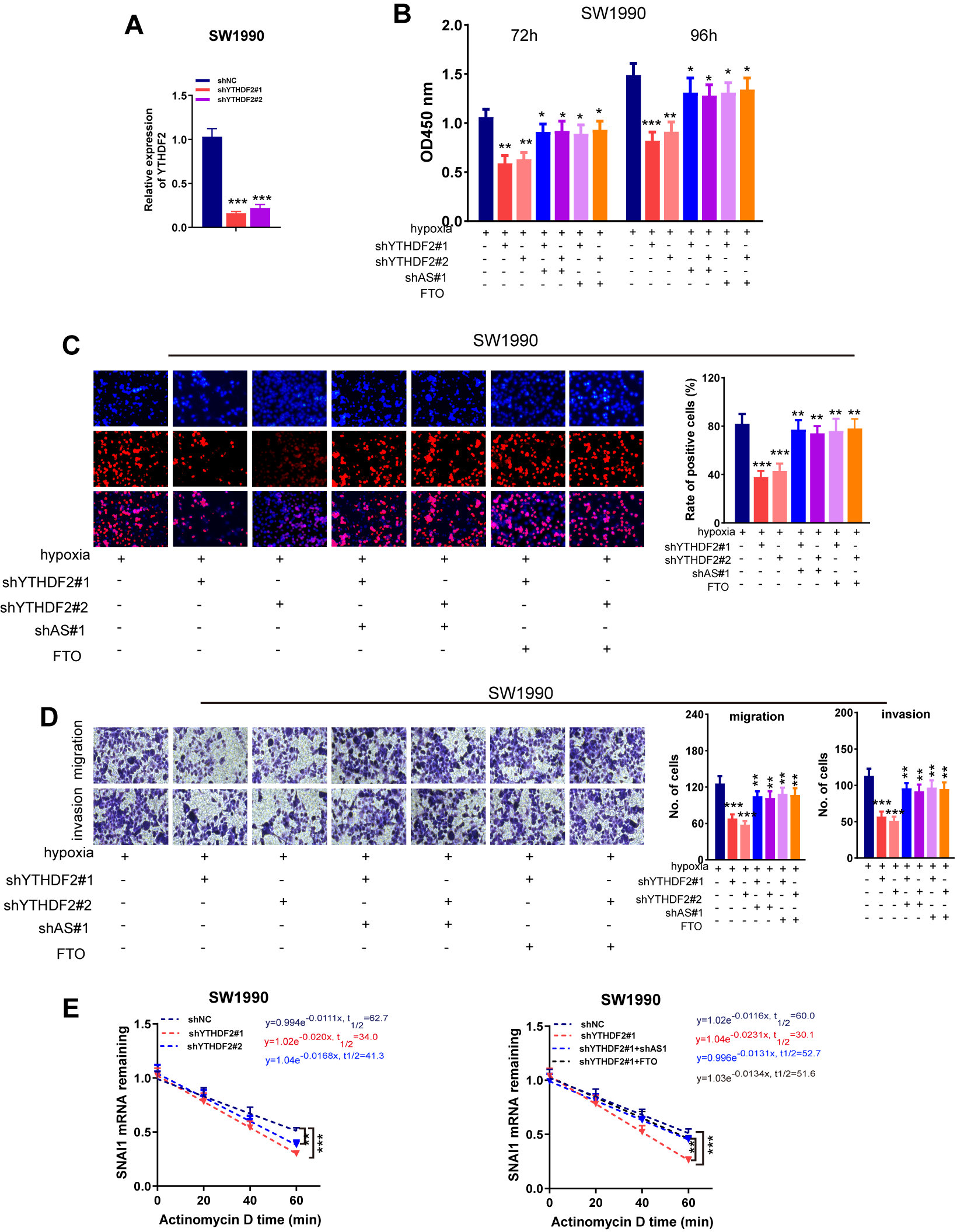

Supplement: Supplementary file 1 — Additional file 1: Table S1. shRNA targeting sequence.Table S2. The sequences of the primers included in this manuscript. Figure S1. Biological characterization of GATA6-AS1. Figure S2. GATA6-AS1 overexpression inhibits EMT process in subcutaneous xenografts from mouse. Figure S3. Association of GATA6-AS1 expression and epithelial–mesenchymal transition markers in human pancreatic ductal adenocarcinoma tissues. Figure S4. GATA6-AS1 knockdown enhances PDAC cells malignant behaviors and EMT.Figure S5. GATA6-AS1 knockdown enhances tumor growth and lung metastasis of PDAC cells. Figure S6. Hypoxia represses GATA6-AS1 expression in PDAC through ETS1. Figure S7. SNAI1 facilitates malignant behaviors and EMT of PDAC cells. Figure S8. FTO is positively correlated with EMT markers in the TCGA-PAAD database. Figure S9. FTO expression in PDAC. Figure S10. YTHDF2 silencing reverses malignant behaviors induced by GATA6-AS1 knockdown in PDAC cells. [file 12967_2023_4757_MOESM1_ESM.doc]
